# Quantum nonlinear spectroscopy of single nuclear spins

**DOI:** 10.1038/s41467-022-32610-8

**Published:** 2022-09-09

**Authors:** Jonas Meinel, Vadim Vorobyov, Ping Wang, Boris Yavkin, Mathias Pfender, Hitoshi Sumiya, Shinobu Onoda, Junichi Isoya, Ren-Bao Liu, J. Wrachtrup

**Affiliations:** 1 3rd Institute of Physics, Research Center SCoPE and IQST, University of Stuttgart, 70569 Stuttgart, Germany; 2 Max Planck Institute for Solid State Research, Stuttgart, Germany; 3 Department of Physics, Centre for Quantum Coherence, and Hong Kong Institute of Quantum Information Science and Technology, The Chinese University of Hong Kong, Shatin, New Territories, Hong Kong, China; 4 College of Education for the Future, Beijing Normal University, Beijing, China; 5 Sumitomo Electric Industries, Ltd., Itami, 664-0016 Japan; 6 Takasaki Advanced Radiation Research Institute, National Institutes for Quantum and Radiological Science and Technology, Takasaki, 370-1292 Japan; 7 Faculty of Pure and Applied Sciences, University of Tsukuba, Tsukuba, 305-8573 Japan

**Keywords:** Quantum metrology, Quantum information

## Abstract

Conventional nonlinear spectroscopy, which use classical probes, can only access a limited set of correlations in a quantum system. Here we demonstrate that quantum nonlinear spectroscopy, in which a quantum sensor and a quantum object are first entangled and the sensor is measured along a chosen basis, can extract arbitrary types and orders of correlations in a quantum system. We measured fourth-order correlations of single nuclear spins that cannot be measured in conventional nonlinear spectroscopy, using sequential weak measurement via a nitrogen-vacancy center in diamond. The quantum nonlinear spectroscopy provides fingerprint features to identify different types of objects, such as Gaussian noises, random-phased AC fields, and quantum spins, which would be indistinguishable in second-order correlations. This work constitutes an initial step toward the application of higher-order correlations to quantum sensing, to examining the quantum foundation (by, e.g., higher-order Leggett-Garg inequality), and to studying quantum many-body physics.

## Abstract (web summary)

Signals that look the same from their low-order correlations can often be distinguished by looking at higher-order ones. Here, the authors exploit the sensitivity of quantum nonlinear spectroscopy to fourth-order correlations to identify Gaussian noises, random-phased AC fields, and quantum spins.

## Introduction

All information one can extract about a physical system is essentially the statistics of measurement, quantified by correlations or moments. It is correlations that distinguish different types of noises or fluctuations. Higher-order correlations are particularly important since different types of physical quantities often have similar or only quantitatively different first- and second-order correlations^1–3^. For example, all the higher order correlations of Gaussian noises can be factorized into first- or second-order correlations of all possible partitions^4^, and those of symmetric dichotomous telegraph noises can be factorized into second-order correlations only in sequential partitions^5^. Higher order correlations are recently used to study many-body physics in cold atom systems^6^ and to reveal the non-Gaussian fluctuations^7^. Measuring correlations of fluctuations in physical systems is important to quantum science and technology. The second-order correlation^8–11^ has enabled high spectral resolution (1–100s Hz) in atomic NMR^11–14^, using nitrogen-vacancy (NV) centers in diamond^15^. Correlations of measurements can test quantum foundations (such as Bell inequality^16^ and Leggett–Garg inequality^17^) and identify the fundamental difference between classical and quantum systems^18–20^.

Nonlinear spectroscopy^21^ is the most widely used approach to determining correlations of fluctuations in a physical system. However, conventional nonlinear optical spectroscopy^21^ and magnetic resonance spectroscopy^22,23^, which use classical probes such as electromagnetic waves, can only access certain types of correlations in a quantum system^24^. The idea of quantum nonlinear spectroscopy^25^ was recently proposed to use quantum probes such as entangled photons to achieve sensitivities and resolutions beyond the classical limits^26,27^. It is shown^28^ that quantum sensing can extract arbitrary types and orders of correlations in a quantum system by first quantum-entangling a sensor and the object and then measuring the sensor^1,29^. Quantum sensing^30^ has been applied to achieve nuclear magnetic resonance (NMR) of single atoms^31–33^ and the second-order correlation spectroscopy^8–11^ has been adopted to enhance the spectral resolution^11–14^. However, quantum nonlinear spectroscopy (i.e., the measurement of higher-order correlations) of single nuclear spins^28^ is still elusive.

Quantum quantities, being operators, usually do not commute, i.e., two quantities $\hat{A}$ and $\hat{B}$ may have a non-zero commutator $[\hat{A},\, \hat{B}]\equiv \hat{A}\hat{B}-\hat{B}\hat{A}\,\ne\, 0$, in sharp contrast to classical quantities, whose commutators always vanish. Therefore, quantum systems have characteristic quantum correlations, which involve commutators of quantities, such as the second-order one $\langle [\hat{A},\, \hat{B}]\rangle$ (in which 〈⋯〉 means the average over many repeated measurements) and the third-order example $\langle \{\hat{A},[\hat{B},\hat{C}]\}\rangle$ (where $\{\hat{A},\hat{B}\}\equiv \hat{A}\hat{B}+\hat{B}\hat{A}$ denotes an anti-commutator). Such quantum correlations can be used for the classical-noise-free detection of quantum objects^24^. The classical correlations reduce to the normal products when the operators are replaced with classical quantities—C-numbers, while the quantum one would vanish.

The rich structures of higher-order correlations are largely unexplored due to the limitation of conventional spectroscopy. In conventional nonlinear spectroscopy, a weak classical “force” *f*_*i*_ is applied to a system at different times and/or locations, with a Hamiltonian ${\hat{V}}_{i}={f}_{i}{\hat{B}}_{i}$, and the change of a physical quantity $\hat{A}$ (the response) is measured. After time-dependent perturbation expansion of unitary evolution of the system, the response of a quantum system to the *K*th order of the weak force is determined by a (*K* + 1)th order correlation that involves only commutator, such as $\langle [{\hat{B}}_{1},[{\hat{B}}_{2},\ldots [{\hat{B}}_{K},\, \hat{A}]]]\rangle$ since the evolution of a quantum system is governed by the commutator of the interaction operator and its density operator. The response of a classical system contains only the classical correlation such as 〈*B*_1_ *B*_2_⋯*B*_*K*_*A*〉. The correlations that involve anti-commutators, such as $\langle \{{\hat{B}}_{1},[{\hat{B}}_{2},\{{\hat{B}}_{3},\,\hat{A}\}]\}\rangle$, do not show up in the response of a quantum system to a classical force. Similarly, the noise spectroscopy can also extract limited types of correlations^1,34–41^.

Quantum probes in lieu of classical forces can be utilized to break the limits of conventional spectroscopy. Quantum light spectroscopy (using, e.g., entangled photons) has been demonstrated to have both high spectral and high temporal resolutions^25,27^. Quantum sensing provides a systematic approach to extracting higher-order correlations of arbitrary types^28^. A quantum sensor can establish entanglement with a quantum target, by which a measurement of the sensor constitutes a measurement of the target^1,29^. Specifically, one can perform a sequence of so-called weak measurements of a target by, repeatedly, weakly entangling the sensor with the target and measuring the sensor. By designing the initial state of the sensor and choosing the measurement basis in each shot of measurement, one can extract different types of correlations of the quantum target via statistics of the sequential outputs^28^. In conventional magnetic resonance spectroscopy, one can in principle separate the spin system into a quantum sensor and a target, but since the “sensor” is measured only at the end of a control sequence, the extractable correlations are restricted to those that can be coded by unitary quantum control or non-unitary ones that can be constructed from unitary controls via, e.g., phase cycling.

Here we demonstrate the extraction of fourth-order correlations of single nuclear spins that cannot be measured in conventional nonlinear spectroscopy, using sequential weak measurement^42,43^ via an atomic quantum sensor, namely, a nitrogen-vacancy center in diamond^15^. This first attempt of quantum nonlinear spectroscopy via quantum sensing already leads to non-trivial discoveries. We show that quantum nonlinear spectroscopy provides fingerprint features to identify different types of objects, such as Gaussian noises, random-phased AC fields, and quantum spins, which would be indistinguishable in second-order correlations. The measured fourth-order correlation unambiguously differentiates a single nuclear spin and a random-phased AC field. It also provides a discrete count of the number of spins (similar to the photon-count correlation for determining the number of quantum emitters).

## Results

### Protocol and modeling

**Fig. 1:**
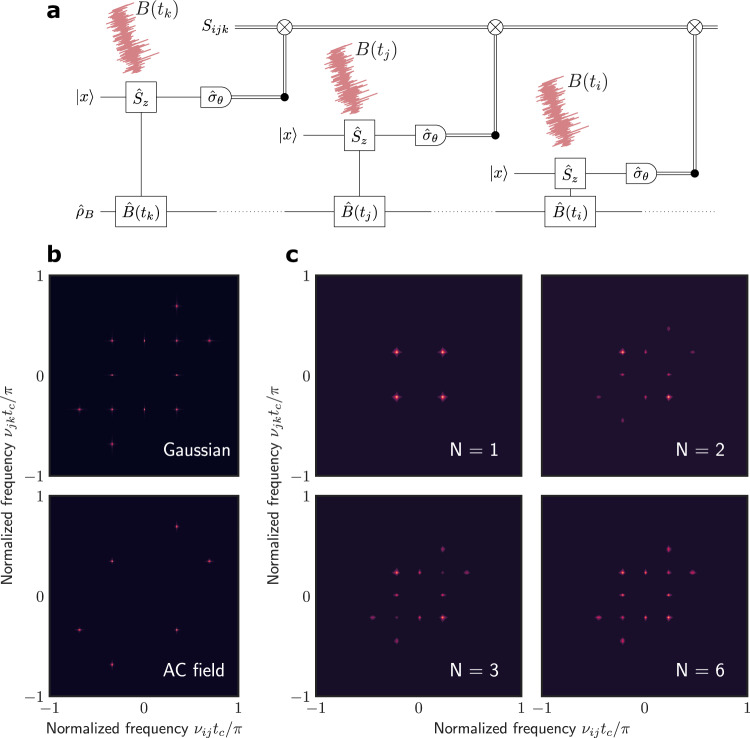
Simulated third-order correlation spectra of different types of classical and quantum noises. **a** The scheme of correlation measurement. A sensor spin is initially prepared in the state |*x*〉, then its *z*-component ${\hat{S}}_{z}$ is coupled to a classical noise *B*(*t*) or a quantum object by $\hat{B}\left(t\right)$, and the measurements along *e*_*θ*_ are correlated to determine the statistical moments, e.g., *S*_*ijk*_. **b** 2D spectra $\widetilde{S}\big({\nu }_{{ij}},{\nu }_{{jk}}\big)$ of the third moment *S*_*ijk*_ for a Gaussian noise (upper) and a random-phased AC field (lower). **c** 2D spectra of the third moment for a sensor coupled uniformly to *N* spin-1/2’s (*N* = 1, 2, 3, and 6).

The sensing protocol is shown in Fig. 1a. In each shot of the sequential weak measurement, we prepare the sensor spin-1/2 in, e.g., the state |*x*〉. We then measure the sensor spin ${\hat{\sigma }}_{\theta }={\hat{\sigma }}_{x}\,{{\cos }}\theta+{\hat{\sigma }}_{y}\,{{\sin }}\theta$ along the direction ***e***_*θ*_ (in the *xy*-plane with an angle *θ* from the *x-*axis). The weak interaction between the sensor and a quantum target $\hat{V}(t)={\hat{S}}_{z}\hat{B}(t)$ (with ${\hat{S}}_{z}$ being the sensor spin along the *z*-axis and $\hat{B}(t)$ the quantum field from the target) can induce weak entanglement in an interrogation time *τ*. The measurement on the sensor spin constitutes a weak measurement of the target. The correlations of the target can be extracted from the statistics of the measurement outputs (*σ*_1_, *σ*_2_,…, *σ*_*j*_,…) with *σ*_*j*_ = ±1. For example, the first moment ${S}_{j}=\langle {\sigma }_{j}\rangle$ was used to detect single nuclear spins^31–33^, and the second moment ${S}_{{ij}}=\langle \delta {\sigma }_{i}{\delta \sigma }_{j}\rangle$ (with $\delta {\sigma }_{i}\equiv {\sigma }_{i}-\langle {\sigma }_{i}\rangle$) was measured for high-resolution atomic NMR^11–14^. Here we concentrate on the third moment ${S}_{{ijk}}=\langle \delta {\sigma }_{i}\delta {\sigma }_{j}\delta {\sigma }_{k}\rangle .$ Not to be confused with the correlations in the targets, the *K*th statistical moment of the measurement outputs will be referred to as the *K*th order “signal”.

Let us first consider a classical noise *B*(*t*) along the *z*-axis. During the interrogation time *τ* in the *j*th shot of measurement, the sensor spin precesses about the *z*-axis by an angle *Φ*_*j*_ ≈ *B*_*j*_*τ* [where *B*_*j*_ ≡ *B*(*t*_*j*_)]. The probability of output *σ*_*j*_ = ±1 of the measurement along *e*_*θ*_ is ${p}_{j}(\pm )=\frac{1\pm {{{{{\rm{cos }}}}}}(\theta -{\Phi }_{j})}{2}$. For short interrogation time *τ* (in comparison to the timescale and the inverse strength of the noise), in the leading orders of coupling strength, the first moment is ${S}_{j}^{{\mathrm {C}}}=\langle {p}_{j}\rangle \approx \,{{{{{\rm{cos }}}}}}\,\theta (1-\langle {\varPhi }_{j}^{2}\rangle /2)$ (where ${p}_{j}\equiv {p}_{j}^{+}-{p}_{j}^{-}$), the second moment ${S}_{{jk}}^{{\mathrm {C}}}=\langle {p}_{j}{p}_{k}\rangle \,\approx \,{{{{{{\rm{sin }}}}}}}^{2}\theta \langle {\varPhi }_{j}{\varPhi }_{k}\rangle$, and the third moment

1 $${S}_{{ijk}}^{{\mathrm {C}}}\approx -\frac{{{{{{{\rm{sin }}}}}}}^{2}\, \theta \,{{{{{\rm{cos }}}}}}\,\theta }{2}\left({{\langle }}\delta {\varPhi }_{i}^{2}{\varPhi }_{j}{\varPhi }_{k}{{\rangle }}+{{\langle }}{\varPhi }_{i}{\delta \varPhi }_{j}^{2}{\varPhi }_{k}{{\rangle }}+{{\langle }}{\varPhi }_{i}{\varPhi }_{j}\delta {\varPhi }_{k}^{2}{{\rangle }}\right),$$

where $\delta {\varPhi }_{j}^{2}\equiv {\varPhi }_{j}^{2}-\langle {\varPhi }_{j}^{2}\rangle$. Here we have assumed that the noise is symmetric and therefore its odd-order correlations vanish. The phase correlations are related to the field correlations by $\langle {\varPhi }_{j}{\varPhi }_{k}\rangle={\tau }^{2}{C}_{jk}^{{\mathrm {C}}}$ and $\langle {\varPhi }_{i}\delta {\varPhi }_{j}^{2}{\varPhi }_{k}\rangle={\tau }^{4}{{\mathrm {C}}}_{{ijjk}}^{{\mathrm {C}}}-{\tau }^{4}{C}_{{ik}}^{{\mathrm {C}}}{C}_{{jj}}^{{\mathrm {C}}}$ with ${C}_{{jk}}^{{\mathrm {C}}}\equiv \langle {B}_{j}{B}_{k}\rangle$ and ${C}_{{ijkl}}^{{\mathrm {C}}}\equiv \langle {B}_{i}{B}_{j}{B}_{k}{B}_{l}\rangle$. The fourth-order correlations may be factorized into second-order ones with pairing patterns characteristic of the noise type. For example, a Gaussian noise allows all pairings, ${C}_{{ijkl}}^{{\mathrm {C}}}={C}_{{ij}}^{{\mathrm {C}}}{C}_{{kl}}^{{\mathrm {C}}}+{C}_{{ik}}^{{\mathrm {C}}}{C}_{{jl}}^{{\mathrm {C}}}+{C}_{{il}}^{{\mathrm {C}}}{C}_{{jk}}^{{\mathrm {C}}},4$ and an AC field with a uniformly random phase has ${C}_{{ijkl}}^{{\mathrm {C}}}=({C}_{{ij}}^{{\mathrm {C}}}{C}_{{kl}}^{{\mathrm {C}}}+{C}_{{ik}}^{{\mathrm {C}}}{C}_{{jl}}^{{\mathrm {C}}}+{C}_{{il}}^{{\mathrm {C}}}{C}_{{jk}}^{{\mathrm {C}}})/2$ (the same as Gaussian noises, except for the factor 1/2) (see Supplementary Note 3). For a noise oscillating with angular frequency *ν*_0_, different types of statistics would yield the same second moment ${S}_{{ij}}^{{\mathrm {C}}}\propto {{\cos }}({\nu }_{0}{t}_{{ij}})$ (with ${t}_{{ij}}\equiv {t}_{i}-{t}_{j}$). But the third moment ${S}_{{ijk}}^{{\mathrm {C}}}$ (which contains the fourth-order correlation of the noise) would have different fingerprint patterns in its 2D spectrum ${\widetilde{S}}^{{\mathrm {C}}}({\nu }_{{ij}},\,{\nu }_{{jk}})$ (obtained by 2D Fourier transform in *t*_*ij*_ and *t*_*jk*_) for different types of noises (see Fig. 1b). In particular, the Gaussian noise has 12 peaks of equal height at (0, ±*ν*_0_), (±*ν*_0_, 0), ±(*ν*_0_, *ν*_0_), ±(*ν*_0_, −*ν*_0_), ±(*ν*_0_, 2*ν*_0_), and ±(2*ν*_0_, *ν*_0_), and the random-phased AC field has six peaks at ±(2*ν*_0_, *ν*_0_), ±(*ν*_0_, −*ν*_0_), and ±(*ν*_0_, 2*ν*_0_) (see Supplementary Note 3).

The key difference between a quantum noise and a classical one is that in the interaction $\hat{V}={\hat{S}}_{z}\hat{B}(t)$ the noise $\hat{B}(t)$ is an operator of the target (see Fig. 1a). In the *j*th shot of measurement, starting from the initial state $\hat{\rho }({t}_{j})={{\hat{\rho }}_{{\mathrm {B}}}({t}_{j})\otimes \hat{\rho }}_{{\mathrm {S}}}$ (where ${\hat{\rho }}_{{\mathrm {B/S}}}({t}_{j})$ is the target/sensor state), the interaction leads to $\hat{\rho }({t}_{j}+\tau)=\hat{\rho }({t}_{j})+\frac{\tau }{i}[\hat{V}({t}_{j}),\hat{\rho }({t}_{j})]+\frac{{\tau }^{2}}{2{i}^{2}}[\hat{V}({t}_{j}),[\hat{V}({t}_{j}),\hat{\rho }({t}_{j})]]+\cdots$. To separate the effects on the sensor and those on the target, the commutator can be decomposed as $-i [\hat{V}({t}_{j}),\hat{\rho }({t}_{j})]=-i[{\hat{S}}_{z},{\hat{\rho }}_{{\mathrm {S}}}]\otimes \frac{1}{2}\{{\hat{B}}_{j},{\hat{\rho }}_{{\mathrm {B}}}({t}_{j})\}+\{{\hat{S}}_{z},{\hat{\rho }}_{{\mathrm {S}}}\}\otimes \frac{1}{2i}[{\hat{B}}_{j},{\hat{\rho }}_{{\mathrm {B}}}({t}_{j})]\equiv 2{S}_{z}^{-}{\hat{\rho }}_{{\mathrm {S}}}\otimes {B}_{j}^{+}{\hat{\rho }}_{{\mathrm {B}}}+2{S}_{z}^{+}{\hat{\rho }}_{{\mathrm {S}}}\otimes \,{B}_{j}^{+}{\hat{\rho }}_{{\mathrm {B}}}$, where ${B}^{+}\hat{A}\equiv (\hat{B}\hat{A}+\hat{A}\hat{B})/2$ (essentially the anti-commutator) reduces to the normal product if $\hat{B}(t)$ is a classical field and ${B}^{-}\hat{A}\equiv (\hat{B}\hat{A}-\hat{A}\hat{B})/(2i)$ (essentially the commutator) vanishes if $\hat{B}(t)$ is a classical field. By choosing to measure the sensor in the basis of ${S}_{z}^{+}{\hat{\rho }}_{{\mathrm {S}}}$ or ${S}_{z}^{-}{\hat{\rho }}_{{\mathrm {S}}}$, one can select the target evolution driven by the commutator or the anti-commutator, i.e., ${B}_{z}^{-}{\hat{\rho }}_{{\mathrm {B}}}$ or ${B}_{z}^{+}{\hat{\rho }}_{{\mathrm {B}}}$, respectively. Therefore, quantum correlations that contain a nested sequence of commutators and anti-commutators of the noise operators can be extracted. Considering a target (such as a nuclear spin) at high temperature, i.e., ${\hat{\rho }}_{{\mathrm {B}}}$ being a constant, the second-order quantum correlation ${{\mathrm {Tr}}}({B}_{j}^{+}{B}_{i}^{-}{\hat{\rho }}_{{\mathrm {B}}})$ vanishes. The third momentum has both classical and quantum contributions, ${S}_{{ijk}}={S}_{{ijk}}^{{\mathrm {C}}}+{S}_{{ijk}}^{{\mathrm {Q}}}$.

The classical part ${S}_{{ijk}}^{{\mathrm {C}}}$ is the same as for classical noises (see the “Methods” section and Supplementary Note 4), except that the products of classical variables should be replaced with anti-commutators such as (*t*_l_ > *t*_*k*_ > *t*_*j*_ > *t*_*i*_)

2 $${C}_{{ijkl}}^{{\mathrm {C}}}={{\mathrm {Tr}}}\left({B}_{l}^{+}{B}_{k}^{+}{B}_{j}^{+}{B}_{i}^{+}{\hat{\rho }}_{{\mathrm {B}}}\right).$$

It should be noted that though the classical correlation in the quantum object takes the same form as in a classical noise, it has a fundamentally different origin. The correlations in the quantum object stem from the back-action of the weak measurement by the sensor, which results from the weak entanglement and measurement of the sensor on the basis of ${S}_{z}^{-}{\hat{\rho }}_{{\mathrm {S}}}$. Importantly, the classical correlation ${C}_{{ijjk}}^{{\mathrm {C}}}$ of a *quantum* object in Eq. (2) does not contribute to conventional nonlinear spectroscopy using a *classical* probe.

The quantum part ${S}_{{ijk}}^{{\mathrm {Q}}}=-\frac{1}{2}{{{{{{\rm{sin }}}}}}}^{2}\theta \,{{{{{\rm{cos }}}}}}\,\theta {\tau }^{4}{C}_{{ijjk}}^{{\mathrm {Q}}}$ (see the “Methods” section). For ${\hat{\rho }}_{{\mathrm {B}}}$ being a constant, the quantum correlation has only one non-vanishing term (*t*_*k*_ > *t*_*j*_ > *t*_*i*_ assumed, see the “Methods” section for details)

3 $${C}_{{ijjk}}^{{\mathrm {Q}}}={{\mathrm {Tr}}}\left({B}_{k}^{+}{B}_{j}^{-}{B}_{j}^{-}{B}_{i}^{+}{\hat{\rho }}_{{\mathrm {B}}}\right).$$

The importance of quantumness lies in the fact that without the heralded polarization of the target by back-action from measurement at *t*_*i*_, the commutators at *t*_*j*_ would vanish^24,28^.

When the quantum object is a two-level system (such as spin-1/2 of ^13^C in diamond), the quantum correlations will double the third moment since ${S}_{{ijk}}^{{\mathrm {Q}}}={S}_{{ijk}}^{{\mathrm {C}}}$ in this case (see the “Methods” section). When the sensor is coupled to multiple (*N*) spin-1/2’s at high temperature (see the “Methods” section), the classical correlation scales as ${C}_{{ijjk}}^{{\mathrm {C}}}\sim {N}^{2}$, and the quantum correlation ${C}_{{ijjk}}^{{\mathrm {Q}}}\sim N$ (since the commutators between different spins vanish). With increasing the number, the quantum spins approach to a classical noise, with Gaussian statistics (resulting from the summation of many independent binary quantities). Figure 1c shows qualitatively different patterns in the correlation spectra of a different number of spin-1/2’s with uniform coupling.

### Measurement of correlations

**Fig. 2:**
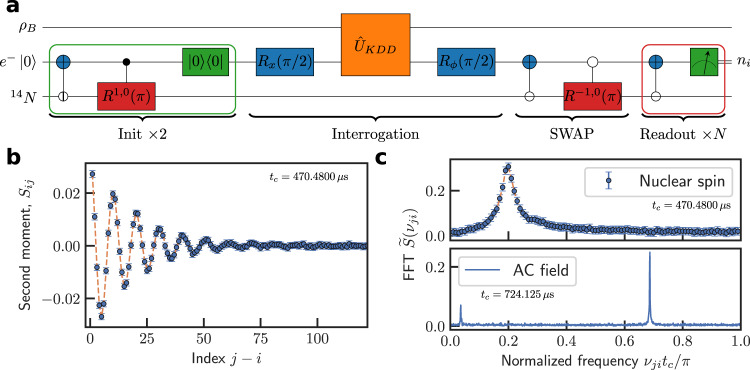
Statistics of sequential measurements on a sensor spin. **a** Protocol of sequential measurement. The sensor spin and the ancilla are initialized by an optical pump (green block being a pulse of 532 nm laser) and SWAP gates (repeated twice for higher fidelity). Then the sensor spin is rotated by a $\frac{\pi }{2}$ pulse (blue block), controlled by a dynamical decoupling sequence, and rotated again by a $\frac{\pi }{2}$ pulse (with a readout angle *θ* from the first $\frac{\pi }{2}$ pulse so that ${\hat{\sigma }}_{\theta }$ is measured). The NV electron spin state is then stored in the ^14^N spin by a SWAP gate and the ^14^N spin state is repeatedly read out through the electron spin via a CNOT gate and photon counts. **b** Second moment *S*_*ij*_ of sequential measurement of a sensor spin coupled to a ^13^C nuclear spin. **c** Fourier transform of the second moment for a nuclear spin (upper) and a random-phased AC field (lower). The extra small peak at lower frequency in lower graph of **c** is not from the AC field, as checked by the dependence of its amplitude on the measurement direction *e*_*θ*_ (see Fig. S5 in Supplementary Note 9).

We employ the states $\vert+\rangle=\vert {0}_{e}\rangle$ and $|-\rangle \equiv|{-1}_{e}\rangle$ in the spin triplet of an NV center in diamond as the sensor spin^15^. Each shot of weak measurement is realized by the pulse sequence shown in Fig. 2a. We optically pump the NV center spin into the state |+〉 and prepare it into the state $|x\rangle=( \vert+\rangle+\vert -\rangle )/\sqrt{2}$ by a $\frac{\pi }{2}$ microwave pulse. A sequence of Knill dynamical decoupling XY (KDD-XY5) consisting of *N*_p_ = 100 pulses modulates the interaction between the NV spin and a target ^13^C nuclear spin during the interrogation such that weak, tuneable entanglement between the sensor and the target is induced. This results in alpha = 0.189 pi interaction strength. The inter pulse time is 186.68 ns, including 68.67 ns for the pi pulse length. Measurement of ${\hat{\sigma }}_{\theta }$ is realized by a $\frac{\pi }{2}$ rotation changing the *e*_*θ*_ axis to the *z*-axis followed by a projective measurement along the *z*-axis. To enhance the readout fidelity, we use a SWAP gate to store the sensor spin state in the ^14^N nuclear spin (which has been polarized in the initialization step using SWAP gates as well) and repeatedly (*M* times) read out the ^14^N spin via a CNOT gate and spin-dependent fluorescence of the NV center electron spin^44,45^. The statistical moments of the measurement *S*_*i*_, *S*_*ij*_, and *S*_*ijk*_ are reconstructed from the photon counts (see the “Methods” section).

Figure 2b shows the second-order signal *S*_*ij*_ of a sensor coupled to a ^13^C nuclear spin. Under an external magnetic field (*B*_0_ = 0.2502 T) along the *z* direction (the NV axis) and dynamical decoupling control of the hyperfine interaction, the quantum field from the ^13^C spin in the interaction picture is effectively $\hat{B}(t)={A}_{\perp }[{\hat{I}}_{x}{{\cos }}({\nu }_{0}t)-{\hat{I}}_{y}{{\sin }}\,({\nu }_{0}t)]$ (see the “Methods” section) with the nuclear Zeeman frequency $\frac{{\nu }_{0}}{2\pi }\,\approx \,2.6795$ MHz. Therefore, ${S}_{{ij}}\propto {C}_{{ij}}^{C}=\frac{1}{2}\langle \{{\hat{B}}_{i}{,\hat{B}}_{j}\}\rangle \propto {{\cos }}({\nu }_{0}{t}_{{ij}}){e}^{-\gamma {t}_{{ij}}}$, oscillates at frequency *ν*_0_ with a measurement-induced decay^9^ (a rapid decay due to random hopping of the NV center state has been removed—see Supplementary Note 9). For comparison, Fig. 2c shows both the Fourier transform of the second-order signal for an AC field $B(t)={B}_{0}{{\cos }}({\nu }_{0}t+\phi )$ with a uniformly random phase *ϕ* and that for a ^13^C nuclear spin. As shown in Fig. 2c, the nuclear spin and the random-phased AC field lead to similar second-order signals.

**Fig. 3:**
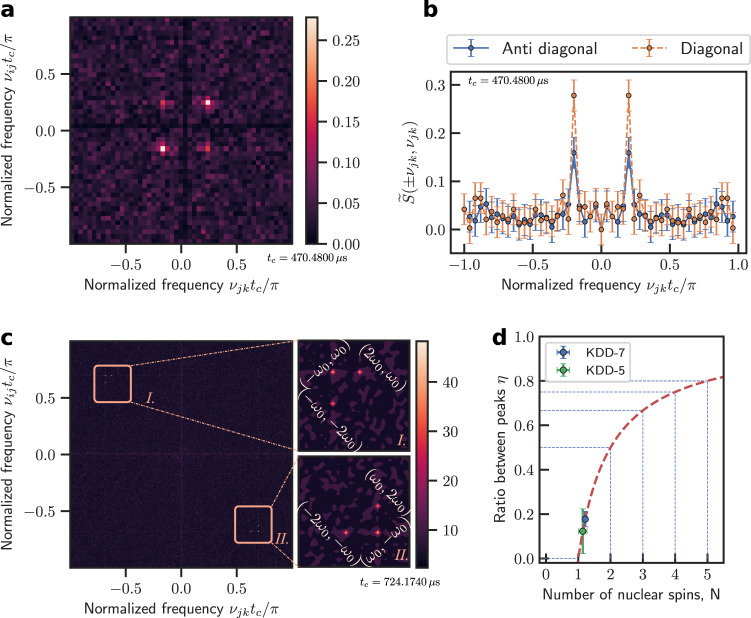
Quantum nonlinear spectroscopy of a nuclear spin and a random-phased AC field. **a** 2D spectrum of the third moment $\widetilde{S}\big({\nu }_{{ij}},{\nu }_{{jk}}\big)$ of an NV center spin coupled to a nuclear spin. **b** The diagonal (orange symbols) and anti-diagonal (purple symbols) slice of **a**. **c** 2D spectrum of the third moment $\widetilde{S}\big({\nu }_{{ij}},{\nu }_{{jk}}\big)$ of an NV center spin coupled to a random-phased AC field. **d** The calculated average height (curve) of the eight peaks at (0, ±*ν*_0_), (±*ν*_0_, 0), ±(*ν*_0_, 2*ν*_0_), or ±(2*ν*_0_, *ν*_0_) relative to those at ±(*ν*_0_, −*ν*_0_) as a function of the number of uniformly coupled nuclear spins. The symbols are experimental values (green is from Fig. 3a and blue is from Supplementary Fig. S12, measured with a different number of dynamical decoupling pulses). Error bars are standard deviation.

The third moment of the sequential measurements has qualitatively different patterns for a quantum spin and for a classical field. We set the measurement angle *θ* ≈ 54.0037° to maximize the amplitude of the third-order signal (which is ∝sin^2^ *θ* cos *θ*). The 2D spectrum of the third moment for a quantum spin target (Fig. 3a, b) clearly shows four peaks at (*ν*_*ij*_, *ν*_*jk*_) with $|{\nu }_{{ij}} \vert=\vert {\nu }_{{jk}} \vert={\nu }_{0}$ mod (2*π*/*t*_c_) with *t*_c_ being the period of each measurement shot. The difference in the heights of the diagonal and anti-diagonal peaks is probably due to the fast hopping between different states of sensor spin (see Supplementary Note 6). In contrast, the 2D spectrum for the random-phased AC field (Fig. 3c), as expected, presents six peaks at (*ν*_*ij*_, *ν*_*jk*_) = ±(*ν*_0_, 2*ν*_0_), ±(*ν*_0_, −*ν*_0_), and ±(2*ν*_0_, *ν*_0_) mod (2*π*/*t*_c_).

The quantum nonlinear spectroscopy has qualitatively different patterns for different numbers of nuclear spins (Fig. 1c). In particular, the height of the eight peaks at (0, ±*ν*_0_), (±*ν*_0_, 0), ±(*ν*_0_, 2*ν*_0_), or ±(2*ν*_0_, *ν*_0_) relative to those at ±(*ν*_0_, −*ν*_0_) is a quantized number *η* = 1–1/*N* (see Fig. 3d and Supplementary Note 5), which provides a discrete count of the number of spins (similar to the determination of the number of quantum emitters by the correlation *g*^(2)^ of photon counts). The relative height *η* averaged over the signals at the eight points is about 0.12 ± 0.1 (Fig. 3d), indicating that the target detected by the sensor is a single nuclear spin. Instead of roughly estimating the number of nuclear spins by sensitivity^46^, our method can determine the exact number if the couplings to different spins are of similar strength.

**Fig. 4:**
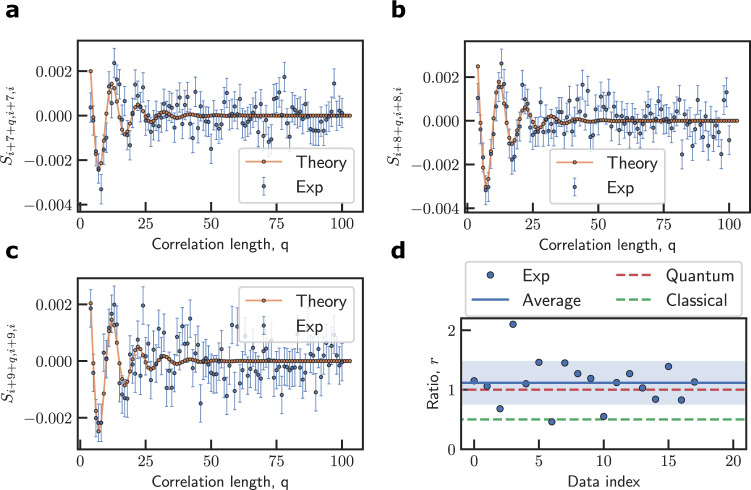
Quantum correlation of a single nuclear spin. **a**–**c** show the third moment *S*_*i*,*i*+*p*, *i*+*p*+*q*_ as a function of *p* for *q* = 7, 8, and 9 in turn. The purple symbols are experimental data. The orange curves are theoretical results with the fitting parameter *r* being the ratio of the amplitude of the third moment to the amplitude squared of the second moment (not shown). *t*_c_ is the same as in Fig. 3a. **d** The factor *r* (purple symbols) obtained from fitting different data sets (see Supplementary Note 11). The blue line is the mean value of *r*, and the shadow area is within one standard deviation from the mean. The red (green) dashed line indicates the value *r*_Q_ = 1 (*r*_C_ = 1/2) for the total (classical only) correlations.

The third moment contains the contribution of the quantum correlation and hence can differentiate a quantum spin and a classical noise. In particular, the second moments for a spin-1/2 at higher temperature is ${S}_{{jk}}={{{\sin }}}^{2}\theta\,{{\cos }}({\nu }_{0}{t}_{{jk}}){e}^{-\gamma {t}_{{jk}}}$ (with *c*_0_ being a constant), the third-order signal for a quantum spin target is ${S}_{{ijk}}=-{\!}r{c}_{0}^{2}\,{{{\sin }}}^{2}\theta \,{{\cos }}\,\theta \,{{\sin }}({\nu }_{0}{t}_{{ij}}){{\sin }}({\nu }_{0}{t}_{{jk}}){e}^{-\gamma {t}_{{ik}}}$ (see the “Methods” section), with *r* = 1 for a quantum spin target and *r*_c_ = 1/2 for the classical signal ${S}_{{ijk}}^{{\mathrm {C}}}$. The fitted result, as shown in Fig. 4d, yield *r* = 1.13 with a standard deviation ≈0.368. The data confirms the quantumness of the noise from the nuclear spin.

## Discussions

The results above demonstrate that quantum nonlinear spectroscopy, enabled by measurement via a quantum sensor, can extract correlations of a quantum object that are inaccessible to conventional nonlinear spectroscopy using classical probes and quantum correlations that are missing in classical fields and cannot be retrieved by conventional noise spectroscopy. The higher-order correlations provide fingerprint features for unambiguous differentiation of noises of different kinds and for verification of quantumness.

Fourth–order correlations of a signal originated from the nuclear spin bath, analogous to the *g*^(2)^(0) measurements of photon statistics, allows to resolve the number of nuclear spins and particularly isolate single spins. In this work, we demonstrated this technique in application to a single nuclear spin. With multiple nuclear spins further research is required to understand how unsimilar coupling affects the correlation function, similar to the effect of dissimilarly bright quantum emitters in quantum optical second-order correlation function (*g*^(2)^(0)). In general, a more efficient readout such as the resonant readout technique at low temperatures^47^ would help experiments by improving the readout efficiency and reducing the unwanted decoherence of nuclear spins induced by the green laser light. This could potentially open the way towards experimental observations of many-body phenomena^28^.

Furthermore, the scheme can be generalized, by using, e.g., different initial states of the sensor spin, different measurement bases, higher orders moments, and higher spins as sensors, to extract arbitrary types and orders of correlations. In our current experiments, the measurement basis is fixed in the whole measurement sequence (along the axis with an angle *θ* from the *x-*axis). This protocol is relatively simple and also allows the fourth-order correlations of the quantum target to be extracted through correlating measurement outputs at three times (*t*_*i*_, *t*_*j*_, and *t*_*k*_). Such a configuration for measuring the fourth order correlations is less demanding on the measurement fidelity and the system stability (needed for a long data acquisition time). However, the shortcoming is also obvious. First, the ${{B}_{j}}^{-}$ of the second measurement occur at the same time (namely, ${B}_{j}^{-}{B}_{j}^{-}$), which limits the spectroscopy to be two-dimensional. Second, more importantly, the measurement along a direction between *x* and *y* axes (relative to the axis of the initial state) makes it impossible to fully distinguish the contribution from the quantum and classical correlations. Actually, in our fourth-order signal, the classical and quantum correlations have equal weight. As shown in ref. 28, by choosing a pair of orthogonal axes along which the sensor state is initialized and is measured, one can selectively address the quantum and classical correlation (given by the commutator ${B}_{j}^{-}$ and the anti-commutator ${B}_{j}^{+}$, respectively). Thus, one task for developing quantum nonlinear spectroscopy is to improve the measurement fidelity and the system stability such that in the sequence of weak measurement, the initial state and measurement axis are individually chosen in each shot of measurement and different types of correlations are fully separated. Such improved capability would enable screening of classical noise for ultrasensitive detection of quantum objects^24^ and facilitate the test of quantum foundation^17^ using higher order quantum correlations.

Information made available by quantum nonlinear spectroscopy will be useful for quantum computing (by helping characterize and optimally suppress noises), quantum sensing (by isolating quantum objects from classical noise background), studying quantum many-body physics (by detecting new types of fluctuations in mesoscopic systems), and examining quantum foundation (by testing higher-order Bell inequalities or Leggett–Garg inequalities with fewer, narrower, or even no interpretation loopholes).

## Methods

### Setup and sample

The measurement is carried out with a confocal microscope setup located in a room temperature bore of a superconducting magnet (see Supplementary Fig. S1). The magnet produces a field of 250 mT, aligned parallel to the NV axis, which results in a transition frequency of about 4.1 GHz between |0〉 and |−1〉. The fluorescence light of the NV centers is detected with an avalanche photo diode (APD). The electron and nuclear spins are manipulated with the two channels of microwaves. We have a typical Rabi frequency of 7 MHz for the electron spin at full pulse amplitude.

The diamond sample used is a 2 mm × 2 mm × 80 μm, (111)-oriented polished slice from a ^12^C-enriched (99.995%) diamond crystal^13^. The single NV centers were created by electron irradiation. The typical lifetimes for the NV centers in this slice are ${T}_{2}^{*}\,\approx\, 50$ μs (measured by Ramsey interference) and ${T}_{2}\,\approx\, 300$ µs (measured by spin echo).

For details of the setup and the sample see Supplementary Note 1.

### Measurement method

We use the NV center electron spin as the sensor and the nitrogen nuclear spin as a quantum memory to enhance the sensing. Each shot of measurement consists of three steps: initialization, sensing and readout. The electron spin is optically initialized in state|0〉. The electron spin is prepared with a (*π*/2)-pulse. We sense ^13^C nuclear spins in diamond with a Knill pulse dynamical decoupling sequence^48^, the KDD*xy*, where the time between pulses matches the Larmor frequency of the ^13^C spin of interest. For KDD-XY5 we used *N*_p_ = 100, *α* = 0.189(4)*π*, and estimated *A*_*x*_ = 2*π* · 14.7(3) kHz. The estimated *A*_*z*_ < 2*π* ⋅ 100 Hz, where $\alpha={N}_{{\mathrm {p}}}\cdot \tau \cdot {A}_{x}/\pi$. Therefore, the superposition state of the NV electron spin acquires a phase, conditioned on the ^13^C state. The sensor state gets projected, orthogonal to the preparation pulse, with a phase-shifted MW pulse. Lastly the optical readout of the sensor spin is performed. Here we SWAP the electron spin state and the ^14^N spin state, which is preserved during several laser readouts, enabling single-shot readout^44,45^. To mitigate the effect of decoherence because of the hyperfine interaction with the target ^13^C when the NV center is in the excited states, we limit the readout to 40 repetitions. The SWAP between the electron and the memory spins consists of a weak MW pulse on the electron spin conditional on the ^14^N state (CNOTe, with duration ∼4 μs) followed by a conditional RF-pulse on the memory spin (CNOTn, with duration ∼50 μs) and then another CNOTe. Each readout repetition consists of one CNOTe and a laser pulse (0.3 μs).

### Reconstruction of correlation from photon counts

The probability for sensor collapses to |0〉 (|−1〉) is denoted by *p*(+) (*p*(−)). For weak noise, $(\sigma ){\;}\approx {\;}[1+\sigma \,{{\cos }}(\theta -\Phi )]/2$. The distribution of photon counts of each measurement is

4 $$p\left(n\right)=p\left(n{{{{{\rm{|}}}}}}+\right)p\left(+\right)+p\left(n{{{{{\rm{|}}}}}}-\right)p\left(-\right),$$

where $p({n|}\pm )=\frac{1}{n!}{e}^{-{n}_{\pm }}{n}_{\pm }^{n}$ is the Poisson distribution and *n*_±_ is the average number of photons detected for the spin state |0〉 or |−1〉, respectively. The photon counts can be written as

5 $$n=\underline{n}+\sigma d+{w}_{\sigma }$$

with $\underline{n}=({n}_{+}+{n}_{-})/2$ is the average photon count, *d* ≡ (*n*_+_ –*n*_−_)/2 is the photon count contrast between the two spin states, and *w*_*σ*_ ≡ *n*–*n*_*σ*_ is the intrinsic photon count fluctuation (due to spontaneous emission, APD efficiency, etc.) satisfying the distribution $p({w}_{\sigma })=p({n}_{\sigma }+{w}_{\sigma }|\sigma )$ with zero mean value. The photon count fluctuation $\delta {n}_{i}\equiv {n}_{i}-\langle {n}_{i}\rangle$ is related to the spin signal fluctuation $\delta {\sigma }_{i}\equiv {\sigma }_{i}-\langle {\sigma }_{i}\rangle$ by

6 $$\delta {n}_{i}=\delta {\sigma }_{i}d+{w}_{{\sigma }_{i}},$$

with the first moment of the photon counts being $\langle {n}_{i}\rangle=\underline{n}+\langle {\sigma }_{i}\rangle d \,\approx\, \underline{n}+c\,\cos \theta$. The second and third moments are $\langle \delta {n}_{j}\delta {n}_{i}\rangle={d}^{2}\langle \delta {\sigma }_{j}\delta {\sigma }_{i}\rangle,$ and $\langle \delta {n}_{k}\delta {n}_{j}\delta {n}_{i}\rangle={d}^{3}\langle \delta {\sigma }_{k}\delta {\sigma }_{j}\delta {\sigma }_{i}\rangle,$ respectively, for *i*, *j*, *k* being different. Here we have used the fact that the intrinsic photon count fluctuations ${w}_{{\sigma }_{j}}$ are independent for different shots of measurements.

### Effective Hamiltonian under dynamical decoupling

The evolution during the interrogation in the interaction picture is $\hat{U}=\hat{T}{{{{{\rm{exp }}}}}}(-i{\int }_{t}^{t+\tau }f(u){\hat{V}}_{{hf}}(u){{\mathrm {d}}u})={{{{{\rm{exp }}}}}}(-i\hat{V}\tau )$, where *f*(*u*) is the modulation function alternating between +1 and −1 due to the dynamical decoupling sequence^4^ and ${\hat{V}}_{{{\mathrm {hf}}}}(u)$ is the hyperfine interaction in the interaction picture. By Magnus expansion for short period of time, the effective coupling $\hat{V}(t)\,\approx\, {\tau }^{-1}{\int }_{t}^{t+\tau }f(u){\hat{V}}_{{{\mathrm {hf}}}}(u){{\mathrm {d}}u}$. For the coupling to a single ^13^C spin, ${\hat{V}}_{{{\mathrm {hf}}}}(t)={A}_{x}{\hat{S}}_{z}{\hat{I}}_{x}(t)$ with ${\hat{I}}_{x}(t)={\hat{I}}_{x}{{\cos }}({\nu }_{0}t)-{\hat{I}}_{y}\,{{\sin }}({\nu }_{0}t)$. Under the KDD, the effective coupling becomes $\hat{V}(t)\,\approx\, {A}_{\perp }{\hat{S}}_{z}{\hat{I}}_{x}(t)$^13^ with ${A}_{\perp }=2{A}_{x}/\pi$.

### Quantum correlations

The relation between the statistics (moments) of the sequential measurement and the correlation of the noise field $\hat{B}(t)$ can be directly obtained by the perturbative expansion of the evolution during interrogation time *τ*. We assume that the bath evolves freely between two adjacent interrogation processes. As shown in Fig. 1a, the measurements at different times, though conducted on a single NV center spin in the experiment, can be viewed as performed independently on different sensor spins $\{{\hat{S}}_{j}\}$, each interacting with the target with Hamiltonian ${\hat{V}}_{j}={\hat{S}}_{j,z}{\hat{B}}_{j}$ from *t*_*j*_ to *t*_*j*_ + *τ*. The initial state of the target and the sensors can be written as ${\hat{\rho }=\hat{\rho }}_{B}\otimes {\hat{\rho }}_{1}\otimes {\hat{\rho }}_{2}\otimes \cdots$ with ${\hat{\rho }}_{j}={|x}\rangle \langle {x|}={\hat{S}}_{j,x}+\frac{1}{2}$ for the *j*th sensor spin and ${\hat{\rho }}_{B}={2}^{-N}$ being the density operator of *N* nuclear spins at high temperature. The evolution due to the interaction with the *j*th sensor can be expanded as

7 $$\hat{\rho }\left(\tau \right)=\hat{\rho }+\frac{\tau }{i}\left[{\hat{V}}_{j},\, \hat{\rho }\right]+\frac{1}{2!}{\left(\frac{\tau }{i}\right)}^{2}\left[{\hat{V}}_{j},\left[{\hat{V}}_{j},\, \hat{\rho }\right]\right]+\cdots$$

The first moment of the measurement is

8 $${S}_{j}={{\langle }}{\hat{\sigma }}_{j,\theta }{{\rangle }}={\cos }\, \theta -\frac{1}{2!}{\tau }^{2}{{{\mathrm {Tr}}}}_{{\mathrm {S}}}\left({\hat{\sigma }}_{j,\theta }\left[{\hat{S}}_{j,z},\left[{\hat{S}}_{j,z},\,{\hat{\rho }}_{j}\right]\right]\right){{{\mathrm {Tr}}}}_{{\mathrm {B}}}\left({B}_{j}^{+}{B}_{j}^{+}{\hat{\rho }}_{B}\right)+\cdots \\={\cos }\theta \left(1-\frac{1}{2}{\tau }^{2}{C}_{{jj}}^{C}+\cdots \right).$$

The second moment (for *t*_*j*_ > *t*_*k*_) is ${S}_{{jk}}=\langle {\delta \hat{\sigma }}_{j,\theta }\delta {\hat{\sigma }}_{k,\theta }\rangle$, where $\delta {\hat{\sigma }}_{j,\theta }\equiv {\hat{\sigma }}_{j,\theta }-\langle {\hat{\sigma }}_{j,\theta }\rangle$. Since in the zeroth order of the fluctuation $\langle \delta {\hat{\sigma }}_{j,\theta }\rangle=0$, the second moment must contain at least one order of the noise field at each time. Thus, in the leading order of the noise field, the second moment is

9 $${S}_{{jk}}\,\approx\, \frac{{\tau }^{2}}{{i}^{2}}{{\mathrm {Tr}}}\left(\delta {\hat{\sigma }}_{j,\theta }\left[{\hat{S}}_{j,z},\,{\hat{\rho }}_{j}\right]\right){{\mathrm {Tr}}}\left(\delta {\hat{\sigma }}_{k,\theta }\left[{\hat{S}}_{k,z},\,{\hat{\rho }}_{,S}\right]\right){{\mathrm {Tr}}}\left({B}_{j}^{+}{B}_{k}^{+}{\hat{\rho }}_{B}\right)={\tau }^{2}\,{{\sin }}^{2}\,\theta {C}_{{jk}}^{C}.$$

The third moment ${S}_{{ijk}}=\langle \delta {\hat{\sigma }}_{i,\theta }{\delta \hat{\sigma }}_{j,\theta }\delta {\hat{\sigma }}_{k,\theta }\rangle$ can be similarly obtained as

10 $${S}_{{ijk}}\,\approx\, -\frac{{\tau }^{4}\,{\cos }\,\theta \,{{\sin }}^{2}\,\theta }{2}\left({C}_{{iijk}}^{{\mathrm {C}}}-{C}_{{ii}}^{{\mathrm {C}}}{C}_{{jk}}^{{\mathrm {C}}}+{C}_{{ijjk}}^{{\mathrm {C}}}-{C}_{{ik}}^{{\mathrm {C}}}{C}_{{jj}}^{{\mathrm {C}}}+{C}_{{ijkk}}^{{\mathrm {C}}}-{C}_{{ij}}^{{\mathrm {C}}}{C}_{{kk}}^{{\mathrm {C}}}+{C}_{{ijjk}}^{{\mathrm {Q}}}\right).$$

See Supplementary Note 4 for details.

### Correlations of *N* uniformly coupled nuclear spins

The noise field from *N* uniformly coupled nuclear spins $\{{\hat{I}}_{n}\}$ can be written as $\hat{B}(t)={\sum }_{n=1}^{N}\,{A}_{\perp }[{\hat{I}}_{n,x}\,{{\cos }}({\nu }_{0}t)+{\hat{I}}_{n,y}{{\sin}}({\nu }_{0}t)]$ in the interaction picture. With ${\hat{\rho }}_{B}={2}^{-N}$, the second-order classical correlation is

11 $${C}_{{jk}}^{{\mathrm {C}}}={{\mathrm {Tr}}}\left[{B}_{j}^{+}{B}_{k}^{+}{\hat{\rho }}_{{\mathrm {B}}}\right]=\frac{1}{4}N{A}_{\perp }^{2}{\cos }\left({\nu }_{0}{t}_{{ij}}\right){e}^{-\gamma {t}_{{jk}}}\sim O(N),$$

where decoherence of nuclear spins (due to, e.g., back-action of the weak measurement between *t*_*j*_ and *t*_*k*_) is taken into account as the exponential decay factor (see ref. 13). The fourth-order classical correlation is

12 $${C}_{{ijkl}}^{{\mathrm {C}}}\equiv {{\mathrm {Tr}}}\left({B}_{i}^{+}{B}_{j}^{+}{B}_{k}^{+}{B}_{l}^{+}{\hat{\rho }}_{B}\right)={C}_{{ij}}^{{\mathrm {C}}}{C}_{{kl}}^{{\mathrm {C}}}+\frac{N-1}{N}\left({C}_{{ik}}^{{\mathrm {C}}}{C}_{{jl}}^{{\mathrm {C}}}+{C}_{{il}}^{{\mathrm {C}}}{C}_{{jk}}^{{\mathrm {C}}}\right) \sim O\left({N}^{2}\right),$$

which is the same as for a telegraph noise for *N* = 1 and approaches to the Gaussian noise for *N* ≫ 1. The second-order quantum correlation ${C}_{{ij}}^{{\mathrm {Q}}}=0$ and the fourth order

13 $${C}_{{ijkl}}^{{\mathrm {Q}}}\equiv {{\mathrm {Tr}}}\left({B}_{i}^{+}{B}_{j}^{-}{B}_{k}^{-}{B}_{l}^{+}{\hat{\rho }}_{{\mathrm {B}}}\right)=\frac{1}{16}N{A}_{\perp }^{4}{\sin }\left({\nu }_{0}{t}_{{ij}}\right){\sin }\left({\nu }_{0}{t}_{{kl}}\right){e}^{-\gamma {t}_{{ij}}-\gamma {t}_{{kl}}}\propto N,$$

which is much smaller than the classical correlation for *N* ≫ 1. For *N* = 1, the classical contribution ${S}_{{ijk}}^{{\mathrm {C}}}\propto {C}_{{ijjk}}^{{\mathrm {C}}}-{C}_{{jj}}^{{\mathrm {C}}}{C}_{{ik}}^{{\mathrm {C}}}$ and the quantum one ${S}_{{ijk}}^{{\mathrm {Q}}}\propto {C}_{{ijjk}}^{{\mathrm {Q}}}$ are equal, and the total third moment

14 $${S}_{{ijk}}=2{S}_{{ijk}}^{{\mathrm {C}}}\propto {\sin }\left({\nu }_{0}{t}_{{ij}}\right){\sin }\left({\nu }_{0}{t}_{{jk}}\right){e}^{-\gamma {t}_{{ik}}}.$$

Its 2D Fourier transform $\widetilde{S}({\nu }_{{ij}},{\nu }_{{jk}})$ has four peaks at ±(*ν*_0_, *ν*_0_) and ±(*ν*_0_, −*ν*_0_), with equal height.

## Supplementary information

Supplementary information

## Data Availability

Data supporting the findings of this study are available within the article and its Supplementary information and from the corresponding authors upon request. Data for reproducing the figures in the main text will be available before the publication in a publicly accessible repository with the link 10.18419/darus-3004.

## Code availability

The custom code operating the experimental setup is available upon request from the corresponding author.
