# Supplementary material for: Quantum nonlinear spectroscopy of single nuclear spins
Source: Nat Commun. 2022 Sep 9;13:5318. doi: 10.1038/s41467-022-32610-8 (PMC9463177; doi:10.1038/s41467-022-32610-8)
Supplement: Supplementary file 1 — Supplementary information [file 41467_2022_32610_MOESM1_ESM.pdf]

Supplementary Information for

"Quantum nonlinear spectroscopy of single nuclear spins"

Jonas Meinel,[1,2,*] Vadim V. Vorobyov,[1,*] Ping Wang,[3,4,*] Boris Yavkin,[1] Mathias Pfender,[1] Hitoshi Sumiya,[5] Shinobu Onoda,[6] Junichi Isoya,[7] Ren-Bao Liu,[3,†] and Jörg Wrachtrup[1,2,‡]

[1]*3rd Institute of Physics, IQST and Centre for Applied Quantum Technologies, University of Stuttgart, 70569, Stuttgart, Germany*

[2]*Max-Planck Institute for Solid State Research, Stuttgart, Germany*

[3]*Department of Physics, Centre for Quantum Coherence, and Hong Kong Institute of Quantum Information Science and Technology, The Chinese University of Hong Kong, Shatin, New Territories, Hong Kong, China*

[4]*Colledge of Education for the Future, Beijing Normal University, China*

[5]*Advanced Materials Laboratory, Sumitomo Electric Industries Ltd., Itami 664-0016, Japan*

[6]*Takasaki Advanced Radiation Research Institute, National Institutes for Quantum and Radiological Science and Technology, Takasaki 370-1292, Japan*

[7]*Faculty of Pure and Applied Sciences, University of Tsukuba, Tsukuba 305-8573, Japan*

[*] These authors contributed equally

[†] rbliu@cuhk.edu.hk

[‡] j.wrachtrup@pi3.uni-stuttgart.de

CONTENTS

Supplementary Note 1. Details of measurement setup and sample 3

Supplementary Note 2. The third order signal for classical Gaussian noise 3

Supplementary Note 3. Correlation signals under random-phased AC fields 4

1. Proof of Wick theorem for AC field 4
2. Correlation signal for AC field 5

Supplementary Note 4. Derivation of statistical moments of sequential measurement 5

Supplementary Note 5. Third order signal for independent nuclear spin bath 7

1. The classical part of third order signal for multiple nuclear spin 7
2. The quantum part of third order signal of for multiple nuclear spin 9

Supplementary Note 6. The extrinsic signal resulted from random hopping of sensor states 10

Supplementary Note 7. Signal to Noise Ratio and the time scale to reveal the signal 12

Supplementary Note 8. Drift of photon counting 13

Supplementary Note 9. Data processing of first and second order signal 14

Supplementary Note 10. The effect of random hopping of NV state on the 3nd signal of nuclear spin 15

Supplementary Note 11. The data for quantum correction term 15

Supplementary Note 12. The effect of stability of photon counting on non-linear finger-print of AC field 16

Supplementary Note 13. The effect of Nitrogen nuclear spin on the signal 16

References 18

Supplementary Note 1. Details of measurement setup and sample

The measurement is carried out with a confocal microscope setup located in a room temperature bore of a superconducting magnet (see Supplementary Fig. S1). The magnet produces a field of 250mT, aligned perpendicular to the diamond surface (111) and parallel to the NV axis, which results in a transition frequency of about 4.1 GHz between $|0\rangle$ and $1\rangle$. The confocal objective allows to address single NV centers with green laser light from a laser diode which is coupled into the objective through a wedged mirror. The laser can be switched on/off with a TTL pulse generated from the arbitrary waveform generator (AWG) Keysight 8190A waveform within 10 ns (in comparison with typical readout time of 300 ns and re-polarization time of 1μs). The objective position is controlled with a piezo stage ($100\times100\times200\,\mu m^3$) to find the NV centers in a confocal map. The fluorescence light of the NV centers passes through the same wedged mirror followed by a pinhole (diameter 50 μm) and a long pass filter (650 nm) before detection with an avalanche photo diode (APD). The electron and nuclear spins are manipulated with the two channels of the AWG, where channel 1 is used for microwave manipulation and amplified with a traveling wave tube amplifier (Hughes-Travelling Wave Tube 8010H amplifier) to about 40 dBm, and channel 2 is used for radio frequency manipulation and amplified to 52 dBm with a rf-amplifier (Amplifier Research 150A250). Both channels are combined before creating the driving fields on a coplanar waveguide where the diamond is glued on. We have a typical Rabi frequency of 7 MHz for the electron spin at full pulse amplitude.

The diamond crystal was grown by the temperature gradient method under high-pressure high-temperature conditions at 5.5 GPa and 1350 °{C} using high-purity Fe-Co-Ti solvent and high-purity ^{12}C-enriched solid carbon. The single NV centers were created from intrinsic nitrogen by irradiation with 2 MeV electrons at room temperature with a total fluence of $1.3\times10^{11}cm^2$ and annealed at 1000°C (for 2 h in vacuum).

Supplementary Note 2. The third order signal for classical Gaussian noise

For a classical noise $b(t)$, the third order correlation of the measurements is

$$S^{C}_{ijk} \approx -\frac{\sin^2\theta\cos\theta}{2}\left(\left\langle\delta\Phi_i^2\Phi_j\Phi_k\right\rangle + \left\langle\Phi_i\delta\Phi_j^2\Phi_k\right\rangle + \left\langle\Phi_i\Phi_j\delta\Phi_k^2\right\rangle\right), \tag{S1}$$

where

$$\Phi_i = \int_{t_i}^{t_i+\tau} b(t)dt \propto \tau b(t_i)$$

is the phase accumulated in each measurement cycle, and $\delta\Phi_j^2 \equiv \Phi_j^2 - \left\langle \Phi_j^2 \right\rangle$. Here we have assumed that $b(t)$ is the noise averaged by modulation function due to the dynamical control (KDD in this paper) and that the duration of the measurement τ is much shorter than the noise correlation time.

The third order correlation of the measurements S_{ijk} is determined by the fourth order correlation of the classical noise $b(t)$ (or Φ). For a Gaussian noise, the high order correlations of $b(t)$ are factorized to all possible pairing of second-order correlation (by Wick's theorem).

Therefore,

$$S^{\rm C}_{ijk} = -\sin^2\theta\cos\theta\left(\langle\Phi_i\Phi_k\rangle\left\langle\Phi_j\Phi_k\right\rangle + \left\langle\Phi_j\Phi_k\right\rangle\left\langle\Phi_i\Phi_j\right\rangle + \langle\Phi_i\Phi_k\rangle\left\langle\Phi_i\Phi_j\right\rangle\right).$$

For a monochromatic noise, $\left\langle\Phi_i\Phi_j\right\rangle = \alpha^2\cos\left(\nu_0 t_{ji}\right)$ and hence

$$S^{\rm C}_{ijk} = -\alpha^4\sin^2\theta\cos\theta\left[\cos\left(\nu_0 t_{ki}\right)\cos\left(\nu_0 t_{kj}\right) + \cos\left(\nu_0 t_{ji}\right)\cos\left(\nu_0 t_{kj}\right) + \cos\left(\nu_0 t_{ki}\right)\cos\left(\nu_0 t_{ji}\right)\right], \quad \text{(S2)}$$

in which $t_{ji} \equiv t_j - t_i$.

The 2D Fourier transform yields twelve peaks at $\pm(\nu_0, 2\nu_0)$, $(\pm\nu_0, 0)$, $\pm(\nu_0, \nu_0)$, $\pm(\nu_0, -\nu_0)$, $\pm(2\nu_0, \nu_0)$, and $(0, \pm\nu_0)$ with equal amplitude.

Supplementary Note 3. Correlation signals under random-phased AC fields

1. Proof of Wick theorem for AC field

Here we consider a magnetic field $b(t) = b\cos(\nu_0 t + \phi)$ with frequency ν_0 and a random phase ϕ distributed uniformly in $[0, 2\pi)$. Using the identity $b_n \equiv b(t_n) = \frac{b}{2}\sum e^{is\nu_0 t_n + is_i\phi}$, the high order correlations becomes

$$\begin{aligned}\langle b_1 b_2 \cdots b_n\rangle &= \frac{b^n}{2^n}\left\langle\prod_{i=1}^{n}\left(\sum_{s_i=\pm1} e^{is_i\nu_0 t_i + is_i\phi}\right)\right\rangle \\ &= \frac{b^n}{2^n}\left\langle\sum_{s_1 s_2 \cdots s_n=\pm1} e^{i\sum_{i=1}^n s_i\nu_0 t_i + i\sum_{i=1}^n s_i\phi}\right\rangle\end{aligned}$$

The averaging over ϕ make $\sum_i s_{i=0}$ and hence only even order correlations survives. As a result, we have

$$\langle b_1 b_2 \cdots b_{2n}\rangle = \frac{b^{2n}}{2^{2n}}\sum_{\sum_{i=1}^{2n} s_i=0, s_i=\pm1} e^{i\sum_{i=1}^{2n} s_i\nu_0 t_i} \quad \text{(S3)}$$

Since $\sum_{i=1}^{2n} s_i = 0$, there must be n number $s_i = 1$ while the other are -1. So the summation runs over all possible pairings, that is,

$$\langle b_1 b_2 \cdots b_{2n} \rangle = \frac{b^{2n}}{2^{2n}} \frac{1}{n!} \left(\sum_{s_1 \eta_3 \cdots s_{2n-1} = \pm 1} e^{i s_1 \nu_0 t_{12}} e^{i s_3 \nu_0 t_{34}} \cdots e^{i s_{2n-1} \nu_0 t_{2n-1,2n}} + \text{all other pairings} \right) \tag{S4}$$

where the factor $1/n!$ is introduced to remove the over-counting of different orders of the n pairs.

In particular, the second order correlation becomes

$$\left\langle b_i b_j \right\rangle = b^2 \cos \nu_0 t_{ij}/2 \tag{S5}$$

Completing this summation for arbitrary n and using Eq. (S5), we obtain

$$\langle b_1 b_2 \cdots b_{2n} \rangle = \frac{1}{n!} \left(\langle b_1 b_2 \rangle \langle b_3 b_4 \rangle \cdots \langle b_{2n-1} b_{2n} \rangle + \text{all other pairings} \right) \tag{S6}$$

2. Correlation signal for AC field

The leading order of second order signal is (see main text)

$$S^{\mathrm{C}}_{ij} \approx \sin^2 \theta \left\langle \Phi_i \Phi_j \right\rangle,$$

where $\Phi_j = \sqrt{2}|\alpha| \cos(\nu_0 t_j + \phi')$ and $\alpha = \sqrt{2} b \int_0^\tau f(t) e^{i\nu_0 t} dt/2$. Therefore

$$\langle \Phi_i \Phi_j \rangle = \alpha^2 \cos\left(\nu_0 t_{ij}\right). \tag{S7}$$

Applying Eq. (S6) to the case of four fields, we obtain the third-order signal

$$S^{\mathrm{C}}_{ijk} \approx -\frac{\sin^2 \theta \cos \theta}{4} \alpha^4 \left\{ \cos\left[\nu_0 (2t_{jk} + t_{ij})\right] + \cos\left[\nu_0 (t_{jk} - t_{ij})\right] + \cos\left[\nu_0 (t_{jk} + 2t_{ij})\right] \right\}. \tag{S8}$$

As a result, the 2D spectrum has six peaks at $\pm(2\nu_0, \nu_0)$, $\pm(\nu_0, \nu_0)$, and $\pm(\nu_0, 2\nu_0)$ with equal height. The 2D spectra in Fig. S1 for different parameters are consistent with Eq.(S8).

Supplementary Note 4. Derivation of statistical moments of sequential measurement

As illustrated in Fig. 1a in main text, the sequential measurement can be viewed as performed on a series of independent sensor spins with the initial state

$$\hat{\rho} = \hat{\rho}_B \otimes \hat{\rho}_1 \otimes \hat{\rho}_2 \cdots,$$

with the jth sensor spins $\hat{\mathbf{S}}_j$ coupled to the target in time $[t_j, t_j+\tau]$ with coupling $\hat{V}_j = \hat{S}_{j,z}\hat{B}_j$.

Let $\rho(t_j) \equiv \hat{\rho}_B(t_j) \otimes \hat{\rho}_j$ be the the state of the jth sensor and the target before interrogation, the interaction over time τ leads to the state

$$\hat{\rho}(t_j+\tau) = \hat{\rho}(t_j) - i\tau\left[\hat{V}_j, \hat{\rho}(t_j)\right] + \frac{(-i\tau)^2}{2!}\left[\hat{V}_j, \left[\hat{V}_j, \hat{\rho}(t_j)\right]\right] + \cdots, \tag{S9}$$

The target state conditioned on the measurement of $\{\hat{\sigma}_{j,\theta}\} \equiv \hat{\sigma}_{j,x}\cos\theta + \hat{\sigma}_{j,y}\sin\theta$ is

$$\hat{\rho}_B(t_{j+1}) = \mathrm{Tr}_j\left[\hat{\sigma}_{j,\theta}\hat{\rho}(t_j+\tau)\right]. \tag{S10}$$

Using the decomposition

$$-i\left[\hat{V}_j, \hat{\rho}_B \otimes \hat{\rho}_j\right] = 2\mathbb{B}_j^- \hat{\rho}_B \otimes \mathbb{S}_{j,z}^+ \hat{\rho}_j + 2\mathbb{B}_j^+ \hat{\rho}_B \otimes \mathbb{S}_{j,z}^- \hat{\rho}_j$$

and the initial state $\hat{\rho}_j = \frac{1}{2} + \hat{S}_{j,x}$, we get the target state after the measurement

$$\hat{\rho}_B(t_{j+1}) = \cos\theta\hat{\rho}_B(t_j) + \tau\sin\theta\mathbb{B}_j^+\hat{\rho}_B(t_j) - \frac{\tau^2}{2!}\cos\theta\mathbb{B}_j^+\mathbb{B}_j^+\hat{\rho}_B(t_j) + O\left(\tau^3\right) \equiv \mathbb{P}_j\hat{\rho}_B(t_j)$$

where $\mathbb{P}_j \equiv \cos\theta + \tau\sin\theta\mathbb{B}_j^+ - \frac{\tau^2}{2!}\cos\theta\mathbb{B}_j^+\mathbb{B}_j^+ + O\left(\tau^3\right)$.

The first moment is

$$S_j = \left\langle \hat{\sigma}_{j,\theta} \right\rangle = \mathrm{Tr}_B\left[\mathbb{P}_j\hat{\rho}_B\right] = \cos\theta\left(1 - \frac{\tau^2}{2}C_{jj}^C\right) + O\left(\tau^3\right) \tag{S11}$$

Here we have assumed the noise is symmetric and hence $\mathrm{Tr}_B\left[\hat{B}_j\hat{\rho}_B\right] = 0$.

To calculate the higher order moments, we consider the measurement of the spin fluctuation $\delta\hat{\sigma}_{j,\theta} \equiv \hat{\sigma}_{j,\theta} - \left\langle \hat{\sigma}_{j,\theta} \right\rangle = \hat{\sigma}_{j,\theta} - S_j$. The bath evolution conditioned on the measurement of $\delta\hat{\sigma}_{j,\theta}$ is

$$\delta\hat{\rho}_B(t_{j+1}) = \mathrm{Tr}_j\left[\hat{\sigma}_{j,\theta}\hat{\rho}(t_j+\tau)\right] - S_j\mathrm{Tr}_j\left[\hat{\rho}(t_j+\tau)\right]. \tag{S12}$$

Note that the second term on the r.h.s of the equation above corresponds to the decoherence of the target due to the interaction with the sensor. The condition evolution can be expanded as

$$\delta\hat{\rho}_B(t_{j+1}) = \mathrm{Tr}_j\left[\delta\mathbb{P}_j\hat{\rho}(t_j)\right] = \delta\mathbb{P}_j\hat{\rho}_B(t_j) \tag{S13}$$

with

$$\delta\mathbb{P}_j \equiv \tau\sin\theta\mathbb{B}_j^+ - \frac{\tau^2}{2!}\cos\theta\left(\mathbb{B}_j^+\mathbb{B}_j^+ - C_{jj}^C + \mathbb{B}_j^-\mathbb{B}_j^-\right) + O\left(\tau^3\right).$$

The second moment is

$$S_{ij} = \left\langle \delta\hat{\sigma}_{j,\theta}\delta\hat{\sigma}_{i,\theta} \right\rangle = \mathrm{Tr}_B\left[\delta\mathbb{P}_j\delta\mathbb{P}_i\hat{\rho}_B\right] = \tau^2\sin^2\theta C_{ij}^C + O\left(\tau^4\right).$$

The third moment is

$$S_{ijk} = \left\langle \delta\hat{\sigma}_{k,\theta}\delta\hat{\sigma}_{j,\theta}\delta\hat{\sigma}_{i,\theta}\right\rangle = \mathrm{Tr}_B\left[\delta\mathbb{P}_k\delta\mathbb{P}_j\delta\mathbb{P}_i\hat{\rho}_B\right]$$
$$= -\frac{\tau^4\sin^2\theta\cos\theta}{2}\left(C^C_{iijk} - C^C_{ii}C^C_{jk} + C^C_{ijjk} - C^C_{jj}C^C_{ik} + C^C_{ijkk} - C^C_{ij}C^C_{kk} + C^Q_{ijjk}\right) + O\left(\tau^6\right),$$

with

$$C^Q_{ijjk} \equiv \mathrm{Tr}_B\left[\mathbb{B}^+_k\mathbb{B}^-_j\mathbb{B}^-_j\mathbb{B}^+_i\hat{\rho}_B\right].$$

Here we have used the fact that any commutator has zero trace (thus $\mathrm{Tr}_B\left[\mathbb{B}^-_k\mathbb{B}^-_k\mathbb{B}^+_j\mathbb{B}^+_i\hat{\rho}_B\right] = 0$) and assumed that the target is at infinitely high temperature (thus $\mathrm{Tr}_B\left[\mathbb{B}^+_k\mathbb{B}^+_j\mathbb{B}^-_i\mathbb{B}^-_i\hat{\rho}_B\right] = 0$).

Supplementary Note 5. Third order signal for independent nuclear spin bath

As discussed in the main text, the third order signal can be divided into the classical part S^{C}_{ijk} and quantum part S^{Q}_{ijk} respectively. We discussed these two part respectively

1. The classical part of third order signal for multiple nuclear spin

For independent nuclear spin bath, the effective coupling to the sensor can be written as $\hat{B} = \sum_{\lambda=1}^{N} A_{\lambda,\perp}\hat{I}_{\lambda,x}$ and hence the super-operator $\mathbb{B}^+_i$ corresponding to $B(t_i)$ can be written as

$$\mathbb{B}^+_i \equiv \sum_{\alpha=1}^{N} A_{\lambda,\perp}\mathbb{I}^+_{\lambda,x}(t_i)$$

Here $\hat{I}_{\lambda,x}(t) = e^{i\hat{H}_B t}\hat{I}_{\lambda,x}e^{-i\hat{H}_B t} \equiv \cos\nu_0 t\hat{I}_{\lambda,x} - \sin\nu_0 t\hat{I}_{\lambda,y}$. For example, the fourth order correlation of $\mathbb{B}^+_{\lambda,i}$ has the telegraph structure($l > k > j > i$)

$$\begin{aligned} C^{\mathrm{C}}_{\lambda,ijkl} &\equiv \mathrm{Tr}_{\mathrm{B}}\left[\mathcal{T}\mathbb{B}^+_{\lambda,l}\mathbb{B}^+_{\lambda,k}\mathbb{B}^+_{\lambda,j}\mathbb{B}^+_{\lambda,i}\hat{\rho}_{\mathrm{B}}\right] \\ &= C^{\mathrm{C}}_{\lambda,kl}C^{\mathrm{C}}_{\lambda,ij} \end{aligned} \tag{S14}$$

where $C^{\mathrm{C}}_{\lambda,ij} = \mathrm{Tr}_{\mathrm{B}}\left[\mathcal{T}\mathbb{B}^+_{\lambda,j}\mathbb{B}^+_{\lambda,i}\hat{\rho}_{\mathrm{B}}\right]$ is the second order correlation of $\mathbb{B}^+_{\lambda,i}$

$$C^{\mathrm{C}}_{\lambda,ij} = \frac{A^2_{\lambda,\perp}}{4}\cos\nu_0 t_{ji}$$

The classical part S^{C}_{ijk} is the same to the signal[Eq.(S18)] if the fourth order correlation of classical magnetic field $b_\lambda(t_i)$ is taken to $\tau^4 C^{\mathrm{C}}_{\lambda,ijkl}$ as defined in Eq. (S14). Therefore, we can use the semi-classical method to analyze the classical part S^{C}_{ijk} of the third order signal. We consider

the stochastic process $b(t) = \sum_{\lambda=1}^{N} b_\lambda(t)$ constructed by many independent noise $b_\lambda(t)$, where $b_\lambda(t)$ is the λth noise with the correlation satisfying the properties of Eq. (S14). Under such case, the phase $\Phi_{i\lambda} = \tau b_\lambda(t_i)$ can also be written as

$$\Phi_i = \sum_{\lambda=1}^{N} \Phi_{i\lambda}$$

where $\Phi_{i\lambda}$ is phase induced by the λth noise, which satisfies the following properties(for the time order $i \le j \le k \le l$)

$$\langle \Phi_{i\lambda_1}\Phi_{j\lambda_2}\Phi_{k\lambda_3}\Phi_{l\lambda_4}\rangle = \begin{cases} \langle \Phi_{i\lambda_1}\Phi_{j\lambda_2}\rangle\langle \Phi_{k\lambda_3}\Phi_{l\lambda_4}\rangle & \lambda_1 = \lambda_2 = \lambda_3 = \lambda_4 \\ \langle \Phi_{i\lambda_1}\Phi_{j\lambda_2}\rangle\langle \Phi_{k\lambda_3}\Phi_{l\lambda_4}\rangle & \lambda_1 = \lambda_2 \neq \lambda_3 = \lambda_4 \\ \langle \Phi_{i\lambda_1}\Phi_{k\lambda_3}\rangle\langle \Phi_{j\lambda_2}\Phi_{l\lambda_4}\rangle & \lambda_1 = \lambda_3 \neq \lambda_2 = \lambda_4 \\ \langle \Phi_{i\lambda_1}\Phi_{l\lambda_4}\rangle\langle \Phi_{j\lambda_2}\Phi_{k\lambda_3}\rangle & \lambda_1 = \lambda_4 \neq \lambda_2 = \lambda_3 \\ 0 & \text{other case} \end{cases} \tag{S15}$$

These properties can be further reformulated as the following form

$$\begin{aligned} &\langle \Phi_{i\lambda_1}\Phi_{j\lambda_2}\Phi_{k\lambda_3}\Phi_{l\lambda_4}\rangle \\ &= \delta_{\lambda_1\lambda_2}\delta_{\lambda_3\lambda_4}\langle \Phi_{i\lambda_1}\Phi_{j\lambda_1}\rangle\langle \Phi_{k\lambda_3}\Phi_{l\lambda_3}\rangle \\ &+\delta_{\lambda_1\lambda_3}\delta_{\lambda_2\lambda_4}(1-\delta_{\lambda_1\lambda_2})\langle \Phi_{i\lambda_1}\Phi_{k\lambda_1}\rangle\langle \Phi_{j\lambda_2}\Phi_{l\lambda_2}\rangle \\ &+\delta_{\lambda_1\lambda_4}\delta_{\lambda_2\lambda_3}(1-\delta_{\lambda_1\lambda_2})\langle \Phi_{i\lambda_1}\Phi_{l\lambda_1}\rangle\langle \Phi_{j\lambda_2}\Phi_{k\lambda_2}\rangle \end{aligned} \tag{S16}$$

we insert this identity into the formula of the third order signal[Eq.(S1)] and discuss the three terms one by one.

We take the first term as an example. Inserting Eq. (S16) to the first term of Eq.(S1), we find

$$\begin{aligned} &-\frac{1}{2}\left\langle \Phi_i\Phi_j\delta\Phi_k^2\right\rangle \\ &= \sum_{\lambda_1=1}^{N}\sum_{\lambda_2=1}^{N}(1-\delta_{\lambda_1\lambda_2})\langle \Phi_{i\lambda_1}\Phi_{k\lambda_1}\rangle\langle \Phi_{j\lambda_2}\Phi_{k\lambda_2}\rangle \end{aligned}$$

If the nuclear spin has the same coupling, we have $\langle \Phi_{i\lambda}\Phi_{j\lambda}\rangle = \langle \Phi_i\Phi_j\rangle/N$ which doesn't depends on λ. As a result, the first term becomes

$$-\frac{1}{2}\left\langle \Phi_i\Phi_j\delta\Phi_k^2\right\rangle = -\frac{(N-1)}{N}\langle \Phi_i\Phi_k\rangle\langle \Phi_j\Phi_k\rangle \tag{S17}$$

The other terms can be calculated similarly. As a result, the total signal becomes

$$S^{\mathrm{C}}_{ijk} = -\sin^2\theta\cos\theta\frac{N-1}{N}\langle\Phi_i\Phi_k\rangle\langle\Phi_j\Phi_k\rangle - \sin^2\theta\cos\theta\frac{(N-1)}{N}\langle\Phi_i\Phi_j\rangle\langle\Phi_i\Phi_k\rangle$$
$$-\sin^2\theta\cos\theta\left[\frac{2N-1}{2N}\langle\Phi_j\Phi_k\rangle\langle\Phi_i\Phi_j\rangle - \frac{1}{2N}\langle\Phi_i\Phi_k\rangle\langle\Phi_j\Phi_j\rangle\right]$$

Since the second order correlation $\langle\Phi_i\Phi_j\rangle = N\alpha^2\cos\nu_0 t_{ji}(\alpha = A_\perp\tau/2 = A_x\tau/\pi)$ for homogenous coupling, we have

$$\begin{aligned} S^{\mathrm{C}}_{ijk} &= -\alpha^4\sin^2\theta\cos\theta N(N-1)\cos\nu_0 t_{ki}\cos\nu_0 t_{kj} \\ &-\alpha^4\sin^2\theta\cos\theta\left[\frac{(2N-1)N}{2}\cos\nu_0 t_{ji}\cos\nu_0 t_{kj} - \frac{N}{2}\cos\nu_0 t_{ki}\right] \\ &-\alpha^4\sin^2\theta\cos\theta N(N-1)\cos\nu_0 t_{ji}\cos\nu_0 t_{ki} \end{aligned} \tag{S18}$$

For $N = 1$, this formula reduces to case of single telegraph noise as shown in the main text.

Then 2D Fourier transform gives

$$\tilde{S}^{\mathrm{C}}(\nu_{ij},\nu_{jk}) = -\alpha^4\sin^2\theta\cos\theta\frac{N(N-1)}{4}\sum_{s=\pm1}\left[\delta(\nu_{ij}-s\nu_0)\delta(\nu_{jk}-2s\nu_0) + \delta(\nu_{ij}-s\nu_0)\delta(\nu_{jk})\right]$$
$$-\alpha^4\sin^2\theta\cos\theta\frac{N}{4}\sum_{s=\pm1}\left[\left(N-\frac{3}{2}\right)\delta(\nu_{ij}-s\nu_0)\delta(\nu_{jk}-s\nu_0) + \left(N-\frac{1}{2}\right)\delta(\nu_{ij}+s\nu_0)\delta(\nu_{jk}-s\nu_0)\right]$$
$$-\alpha^4\sin^2\theta\cos\theta\frac{N(N-1)}{4}\left[\delta(\nu_{ij}-2s\nu_0)\delta(\nu_{jk}-s\nu_0) + \delta(\nu_{ij})\delta(\nu_{jk}-s\nu_0)\right]$$

where ν_{ij},ν_{jk} is the frequency corresponding to the variable t_{ij},t_{jk}. There are twelve peaks. For $N = 1$, there are only four peaks, which is the same to the case of single telegraph noise. As N increases to infinity, the twelve peaks has the same height, which is the same to the feature of Gaussian noise.

2. *The quantum part of third order signal of for multiple nuclear spin*

The quantum correction S^{Q}_{ijk} is simplified to

$$\begin{aligned} S^{\mathrm{Q}}_{ijk} &= -\frac{1}{2}\sin^2\theta\cos\theta\sum_{\lambda=1}^{N}\alpha_\lambda^4\mathrm{Tr}\left\{\mathbb{I}^+_\lambda(t_k)\mathbb{I}^-_\lambda(t_j)\mathbb{I}^-_\lambda(t_j)\mathbb{I}^+_\lambda(t_i)\hat{\rho}_{\mathrm{B}}\right\} \\ &= -\frac{1}{2}\sin^2\theta\cos\theta N\alpha^4\sin\nu_0 t_{kj}\sin\nu_0 t_{ji} \end{aligned} \tag{S19}$$

Here we have assume that different nuclear spin has the same coupling.

From Eq.(S18) and Eq. (S19), we find the Fourier transform to be

$$\begin{aligned}\widetilde{S}(\nu_{ij},\nu_{jk}) &= -\alpha^4\sin^2\theta\cos\theta\frac{N(N-1)}{4}\sum_{s=\pm1}\left[\delta(\nu_{ij}-s\nu_0)\delta(\nu_{jk}-2s\nu_0)+\delta(\nu_{ij}-s\nu_0)\delta(\nu_{jk})\right]\\ &-\alpha^4\sin^2\theta\cos\theta\frac{N}{4}\sum_{s=\pm1}\left[N\delta(\nu_{ij}+s\nu_0)\delta(\nu_{jk}-s\nu_0)+(N-2)\,\delta(\nu_{ij}-s\nu_0)\delta(\nu_{jk}-s\nu_0)\right]\\ &-\alpha^4\sin^2\theta\cos\theta\frac{N(N-1)}{4}\left[\delta(\nu_{ij}-2s\nu_0)\delta(\nu_{jk}-s\nu_0)+\delta(\nu_{ij})\delta(\nu_{jk}-s\nu_0)\right]\end{aligned} \tag{S20}$$

The height of the eight peaks $\widetilde{S}(\pm\nu_0,\pm2\nu_0)$, $\widetilde{S}(\pm\nu_0,0)$ $\widetilde{S}(\pm2\nu_0,\pm\nu_0)$, $\widetilde{S}(0,\pm\nu_0)$ is the same to the semi-classical theory but that of the four peaks $\widetilde{S}(\pm\nu_0,\pm\nu_0)$, $\widetilde{S}(\pm\nu_0,\pm\nu_0)$ is corrected by the quantum feedback effect. This ratio

$$\left|\frac{\widetilde{S}(\nu_0,2\nu_0)}{\widetilde{S}(\nu_0,-\nu_0)}\right| = 1-\frac{1}{N}$$

can be used to measure the number of nuclear spin with the same coupling.

Supplementary Note 6. The extrinsic signal resulted from random hopping of sensor states

Expect for intrinsic signal induced by the target, there are some other contributions, for example, the random hopping between different states of the sensor(the charge states or spin states) due to the laser illumination. These hopping can be modeled by a Two Level System(TLS) with its level denotes by $|+\rangle, |-\rangle$. For example, + denotes the charge state NV^- and − denotes charge state NV^0. The dynamics can be described by the following Markovian chain model: The state of TLS can is described by $\mathbf{x}^{(i)} = \{x_-^{(i)}, x_+^{(i)}\}$ with $x_{+(-)}^{(i)}$ denotes the probability of state +(−) at the ith time point. The dynamics of the probability $\mathbf{x}$ abides the following equations

$$\mathbf{x}^{(n+1)} = \mathbf{M}\mathbf{x}^{(n)}$$

where

$$\mathbf{M} = \begin{pmatrix} 1-\eta_b & \eta_d \\ \eta_b & 1-\eta_d \end{pmatrix}$$

is the transition matrix. η_b is the transition probability from $|-\rangle$ to $|+\rangle$ and η_d is the that from + to −. η_b, η_d depends on the details of the physical process. It is easy to check that the equation conserves the population($x_+^{(n)} + x_-^{(n)} \equiv 1$).

In the ith measurement, the electron state would collapse to $|-\rangle$ and $|+\rangle$ with a probability $x_-^{(i)}$ and $x_+^{(i)}$. If the electron state collapse to $|+\rangle$, the outcome is denoted by $u_i = +$ and electron state

collapse to $|-\rangle$ denoted by $u_i = -$. The correlation signal now becomes

$$G_{ij} = \sum_{u_i u_j} u_i u_j p(u_i, u_j) \tag{S21}$$

and third order signal becomes

$$G_{ijk} = \sum_{u_i u_j u_k} u_i u_j u_k p(u_i, u_j, u_k) \tag{S22}$$

Then we give the expression of the joined distribution. The joined distribution of the two outcomes becomes

$$p(u_i, u_j) = p(u_i|u_j)p(u_j)$$

where $p(u_j)$ is the probability of u_j and can be formally written as

$$p(u_j) = \mathrm{Tr}\left[\mathbf{P}_{u_j} \cdot \mathbf{x}^{(j)}\right]$$

The trace here means $\mathrm{Tr}\mathbf{x}^{(i)} = \sum_u x_u^{(i)}$. $\mathbf{P}_u = (1 + u\hat{\sigma}_z)/2(u = \pm)$ is the projection operators and $\hat{\sigma}_z$ is the Pauli matrix

$$\hat{\sigma}_z = \begin{pmatrix} -1 & 0 \\ 0 & 1 \end{pmatrix}$$

It is easy to check that $\mathbf{P}_+ \cdot \mathbf{x}^{(0)} = x_+^{(0)}\{0, 1\}$ and $\mathbf{P}_- \cdot \mathbf{x}^{(0)} = x_-^{(0)}\{1, 0\}$. Hence $\mathrm{Tr}\left[\mathbf{P}_{u_j} \cdot \mathbf{x}^{(j)}\right] = x_{u_j}^{(j)}$ is just the probability for the state be u_j.

Then we calculate the conditional probability $p(u_i|u_j)$. $p(u_i|u_j)$ means the probability of the ith outcome be u_i if the jth measurement give the outcome u_j. If the jth measurement give outcome u_j, the state of the system becomes $\mathbf{P}_{u_j} \cdot \mathbf{x}^{(j)}/\mathrm{Tr}\left[\mathbf{P}_{u_j} \cdot \mathbf{x}^{(j)}\right]$ and the state at time i now becomes $\mathbf{M}^{i-j-1} \cdot \mathbf{P}_{u_j} \cdot \mathbf{x}^{(j)}/\mathrm{Tr}\left[\mathbf{P}_{u_j} \cdot \mathbf{x}^{(0)}\right]$. As a result, $p(u_i|u_j)$ becomes

$$p(u_i|u_j) = \mathrm{Tr}\left\{\mathbf{P}_{u_i} \cdot \mathbf{M}^{i-j-1} \cdot \mathbf{P}_{u_j} \cdot \mathbf{x}^{(j)}\right\} / \mathrm{Tr}\left[\mathbf{P}_{u_j} \cdot \mathbf{x}^{(j)}\right]$$

These result gives the joined distribution

$$p(u_i, u_j) = \mathrm{Tr}\left\{\mathbf{P}_{u_i} \cdot \mathbf{M}^{i-j-1} \cdot \mathbf{P}_{u_j} \cdot \mathbf{x}^{(j)}\right\}$$

If the system is ergodic, the initial state $\mathbf{x}^{(j)}$ is just the steady state of $\mathbf{M}$ and the the joined distribution becomes

$$p(u_i, u_j) = \mathrm{Tr}\left\{\mathbf{P}_{u_i} \cdot \mathbf{M}^{i-j-1} \cdot \mathbf{P}_{u_j} \cdot \mathbf{x}_{ss}\right\} \tag{S23}$$

where $\mathbf{x}_{ss}$ is defined as $\mathbf{M}\cdot\mathbf{x}_{ss} = \mathbf{x}_{ss}$ and $\mathrm{Tr}\mathbf{x}_{ss} = 1$. Generally, the nth order joined distribution can be written as

$$p(u_{i_n}, u_{i_{n-1}}, \cdots, u_{i_1}) = \mathrm{Tr}\left\{\mathbf{P}_{u_{i_n}}\cdot\mathbf{M}^{i_n-i_{n-1}-1}\mathbf{P}_{u_2}\cdots\mathbf{M}^{i_2-i_1-1}\cdot\mathbf{P}_{u_1}\cdot\mathbf{x}_{ss}\right\} \tag{S24}$$

Using the Eq.(S21),Eq.(S22),Eq.(S23) and Eq.(S24), the correlation signal can be reformulated as

$$\begin{aligned} G_i &= \mathrm{Tr}\left[\hat{\sigma}_z\cdot\mathbf{x}_{ss}\right] \\ G_{ij} &= \mathrm{Tr}\left[\hat{\sigma}_z\cdot\mathbf{M}^{i-j-1}\cdot\hat{\sigma}_z\cdot\mathbf{x}_{ss}\right] \\ G_{ijk} &= \mathrm{Tr}\left[\hat{\sigma}_z\cdot\mathbf{M}^{i-j-1}\cdot\hat{\sigma}_z\cdot\mathbf{M}^{j-k-1}\cdot\hat{\sigma}_z\cdot\mathbf{x}_{ss}\right] \end{aligned} \tag{S25}$$

when the summation of u_i is completed

The steady state $\mathbf{x}_{ss}$ can be calculated using the equations

$$\mathbf{M}\mathbf{x}_{ss} = \mathbf{x}_{ss}$$

$$\mathrm{Tr}\mathbf{x}_{ss} = 1$$

the result is

$$\mathbf{x}_{ss} = \frac{\{\eta_d, \eta_b\}}{\eta_d + \eta_b}$$

Using this stable state, direct calculation gives

$$\begin{aligned} G_i &= \frac{\eta_d - \eta_b}{\eta_d + \eta_b} \\ S_{ij} = G_{ij} - G_iG_j &= \frac{4\eta_b\eta_d}{\eta_b + \eta_d}(1-\eta_b-\eta_d)^{i-j-1} \\ S_{ijk} &= -(2-\eta_b-\eta_d)\left(\frac{\eta_b-\eta_d}{\eta_b+\eta_d}\right)\frac{4\eta_b\eta_d}{(\eta_b+\eta_d)^2}(1-\eta_b-\eta_d)^{i-j-1}(1-\eta_b-\eta_d)^{j-k-1} \end{aligned} \tag{S26}$$

Supplementary Note 7. Signal to Noise Ratio and the time scale to reveal the signal

Then the shot noise to reconstruct S_i is

$$\delta S_i = \frac{\Delta}{\sqrt{M}}$$

where M is measurement times of the sequential measurement and Δ is the noise per shot

$$\Delta = \frac{1}{d}\sqrt{\left\langle n_i^2\right\rangle - \langle n_i\rangle^2} = \frac{1}{d}\sqrt{\sum_{u=\pm1} p(u)n_u(n_u+1) - \left(\sum_{u=\pm1} p(u)n_u\right)^2} \tag{S27}$$

where $d = (n_+ - n_-)/2$ and $p(\pm) \approx (1 \pm \cos\theta)/2$. Since δn_i is independent with δn_j, the noise amplitude for second and third order correlation then becomes

$$
\begin{aligned}
\Delta S_{ij} &= \frac{\Delta^2}{\sqrt{M}} \\
\Delta S_{ijk} &= \frac{\Delta^3}{\sqrt{M}}
\end{aligned}
\tag{S28}
$$

For a rough estimation of Δ, we take $p(\pm) \approx 1/2$. ΔS_i is approximately

$$\Delta S_i \approx \frac{1}{d}\sqrt{\frac{(n_+ - n_-)^2 + 2(n_+ + n_-)}{4}} \approx \frac{\sqrt{2(n_+ + n_-)}}{n_+ - n_-} = \frac{1}{\sqrt{\overline{n}}}\frac{1}{C}$$

where C is the contrast of photon fluorescence.

We plot the noise strength as a function of the measurement times in Fig.S3(d). The scatters is the noise amplitude obtained from the trajectory of the photon number via the method show in Fig.S3(c). These results are well consistent with the theoretical result in Eq.(S27) and Eq.(S28) with $C = 0.7$.

We then estimate how many measurement is required to detect the third order signal. After the 2D Fourier transform, the peak value of third order signal is $S_p = 4\sin^2\theta\cos\theta$. The noise strength in the frequency domain is

$$\Delta\widetilde{S} = \left(\sqrt{N_{\mathrm{F}}}\right)^2 \frac{\Delta^3}{\sqrt{M}} = \frac{4R}{\alpha^2}\frac{\Delta^3}{\sqrt{M}}$$

where $N_{\mathrm{F}} = 4R/\alpha^2$ is FFT length to do the Fourier transform and $R \sim 1$ is a constant. Hence the (Signal-to-Noise Ratio)SNR is now

$$\mathrm{SNR} = \sqrt{M}\frac{\sin^2\theta\cos\theta\alpha^2}{R\Delta^3}$$

As a result,

$$M = \frac{\mathrm{SNR}^2 R^2 \Delta^6}{\sin^4\theta\cos^2\theta\alpha^4}$$

Under current experimental condition $\overline{n} \approx 4$ and $C = 0.17$, we have $\Delta \approx 2.9$. Let $\mathrm{SNR} = 4$, $R = 2$ and $\alpha = 0.4$, we estimate $M \approx 10^7$.

Supplementary Note 8. Drift of photon counting

For large interval $j - i$, $\delta n_i, \delta n_j$ should be independent of each other and hence the correlation $\langle \delta n_i \delta n_j \rangle$ must vanish. However, the $\langle \delta n_i \delta n_j \rangle$ is not zero for large interval $j - i$ as shown in Fig. S4(a) and (b). This indicate there is drift for the photon counting, or in other words, the averaged photon number $\langle n \rangle$ is time dependent. As a result, we divide the photon trace to many subgroups

and each subgroup contains 1×10^5 times outputs. In this duration, averaged photon number $\langle n \rangle$ drifts slightly. Then we reconstruct the signal in each subgroup and then average these results for different groups to obtain the final signal.

Due to the drift of photon, we need remove a slow linear decay signal to reduce its effect on the correlation signal. For example, the third order correlation before and after post processing is shown in Fig. S4(c) and (d).

Supplementary Note 9. Data processing of first and second order signal

The random hopping of NV states[Eq.(S26)] will contribute an extra signal to the second correlation signal. We need remove this signal via post processing. This can be achieved by fitting the the total correlation signal through the formula

$$S_{ij} = ae^{-(j-i-1)\gamma} + Ae^{-(j-i-1)\Gamma} \cos n\Theta + b$$

where the first term is signal induced by random hopping of the NV states[Eq. (S26)], the second term is the signal of nuclear spin or AC filed, and the third term is the back ground.

In Fig. S5(c) we show the fitted amplitude A as a function of the readout angle θ for nuclear spin. For AC field, as shown in the main text, there are two peaks in the Fourier transform of the second order signal. One peaks comes from the AC signal while the other comes from unknown noise. This is proved by the dependence of the height of the two peaks on the readout angle as indicated in Fig. S5(d).

In Fig. S6(b), we also show the fitted Θ of nuclear spin signal as a function of the precessing angle $\nu_0 t_c$ in each cycle. It show a linear dependence before a turning point and saturates to π after that points. This is the measurement induced drag effect and has been discussed in detail in Ref. [1].

In Fig. S6(c), we show the fitted decay Γ per cycle as a function of τ(or the KDD number). It shows a linear dependence. Since the theoretical value $\Gamma = \alpha^2/4 + \Gamma_{\rm ex}(\alpha = A_x\tau/\pi)$[1], this linear dependence can be used to measure the transverse coupling A_x between the sensor and nuclear spin. In Fig. S6(d), Γ is independent of θ which is consistent with theory.

Supplementary Note 10. The effect of random hopping of NV state on the 3nd signal of nuclear spin

The random hopping of NV states[Eq.(S26)] will also contribute an extra signal on the third correlation signal. We also remove it by the same method in Sec. Supplementary Note 9. After post processing, we show the third order signal of nuclear spin for different NV and different parameters in Fig. S7 to Fig. S12. As shown in Fig. S7(a) and (b), there is a large deviation between the experimental result and that of theoretical value of S_{ijk} for small $k-j, j-i$. The experimental result is more like the second order signal because there is $\pi/2$ phase shift between experimental theoretical results[see in Fig. S7(a) and (b)]. This indicates this extra signal(the hopping of states of NV center) has not been remove completely in the third signal. However, as increasing of $k-j, j-i$, the experimental results gradually show the same feature with the theoretical result because this extra signal decays exponentially[See Fig. S7(c) and (d)].

Supplementary Note 11. The data for quantum correction term

As the quantum correction increase the signal amplitude by a factor 2[see discussion in the main text], we can testify the existence of quantum correction term[Eq. (S19)] by checking the amplitude of the third signal. Since the absolute signal is difficult to know exactly, we compare the relative amplitude between the second order signal and third order signal. More exactly, we fit the second order signal and third order signal by the formula

$$S_{ij} = c^2 \sin^2\theta\alpha^2 \sin[\nu_0(t_j - t_i)]e^{-(j-i-1)\alpha^2/4}$$
$$S_{ijk} = -rc^3 \sin^2\theta\cos\theta\alpha^4 \sin[\nu_0(t_k - t_j)]e^{-(k-j-1)\alpha^2/4} \sin[\nu_0(t_j - t_i)]e^{-(j-i-1)\alpha^2/4}$$

with only three fitting parameters r, c and α. Since the photon counting model is not know exactly, reconstruction of the correlation signal will bring a factor c^2 and c^3 for second and third order signal respectively. The factor r then quantifies the relative strength between the second and third order signal. As discussed in in the main text, the semi-classical theory predicts $r = 0.5$ while quantum theory predicts $r = 1$. Then the fitted factor r can be used to identify the existence of quantum correction term.

In Fig. S7 to Fig. S12, we plot the third order signal which has better SNR for different sample and different control parameters. The fitted factor r is also labeled in these figures.

Supplementary Note 12. The effect of stability of photon counting on non-linear finger-print of AC field

The drift of the photon counting will introduce residual second order signal to the third order signal. We can use proper post-processing method to remove this residual second order signal. As shown in Fig. S13(a) and (b), we show the non-linear finger-print for different post processing. In Fig. S13(a), we divided the photon trace to many subgroups with each subgroup including 10^5 times outputs. Then we reconstruct the correlations signal and average them for different subgroups. In contrast, in Fig. S13(b), we divide the photon trace to more subgroups with each subgroup including 2.5×10^4 times outputs and the correlations is reconstructed by the same method. These two different post processing lead to non-linear finger-print with slight difference. In Fig. S13(a), the non-linear finger-print shows extra four peaks in the diagonal line when compared with Fig. S13(b). This is because that the length of the subgroup is too long such that the photon counting drifts dramatically and hence the second order signal is not removed clearly via the formula

$$S_{ijk} = \langle \delta n_i \delta n_j \delta n_k \rangle = G_{kji} - \left(G_{kj} G_i + G_{ji} G_k + G_{ki} G_j \right) - G_k G_j G_i$$

These extra four peaks comes from the second order signal of the AC field and another unknown signal(see Fig. 3 in main text). Indeed, the drift of photon counting can be revealed by the photon trace[Fig. S13(c)] and photon correlations[Fig. S13(d)]. These results prove that the four extra peaks indeed comes from improper post processing.

Supplementary Note 13. The effect of Nitrogen nuclear spin on the signal

We further investigate systematic effects due to imperfection of the electron pulse caused by the existence of ^{14}N. ^{14}N has three levels and each level will shift the electron energy level differently due to the hyperfine coupling. Due to the energy shift(2.2MHz) is comparable to the Rabi frequency(7MHz), the Micro Wave can't resonantly excite simultaneously the three transitions associated to the three nitrogen levels. As a result, the existence of nitrogen will introduce spin dependent imperfection to the electron pulse. Due to the KDD sequence is robust to the pulse error, the dominated error comes from the half π pulse applied to the electronic spin. It is this imperfection that causes the signals to dependent on the polarization of nitrogen and the frequency of Micro Wave(MW).

We show the observed first order signal in Fig. S14. When the nitrogen is not fully polarized, it shows an obvious deviation from the prediction under idea case($\propto \cos\theta$)[see Fig. S14(a)] and the experimental curve becomes asymmetric. This can be accounted for by a simplified model as following.

Due to the sequential measurement, the steady state of nitrogen will be driven to the steady state which depends on the imperfection of the electron pulse. Since the first order signal is determined by the steady state of nitrogen, we calculate this steady state in the following. Firstly, we neglect the level $|-1_{\rm N}\rangle$ because the nitrogen has been polarized to the subspace of $|0_{\rm N}\rangle$ and $|1_{\rm N}\rangle$. Then, we assume the steady population of this two spin state is $p^s_{1_{\rm N}}$ and $p^s_{0_{\rm N}}$. So the total state before the first half π pulse can be written as

$$\rho = |0_e\rangle\langle 0_e| \otimes \left(|1_{\rm N}\rangle\langle 1_{\rm N}|p^s_{1_{\rm N}} + |0_{\rm N}\rangle\langle 0_{\rm N}|p^s_{0_{\rm N}}\right) \tag{S29}$$

The two nuclear spin state denotes two path and each path will introduce different imperfection to the the half π pulse. Effectively, the imperfection can be accounted by a spin-dependent modification $\delta\theta_m = 2\delta_m/\Omega(\delta_0 - \delta_1 \equiv 2\delta = 2.2\text{MHz})$ to the readout angle θ. As a result, the density matrix after the second half π pulse becomes(neglected the irrelevant coherence)

$$\begin{aligned}\rho = p^s_{1_{\rm N}}&\left[\frac{1+\cos(\theta+\delta\theta_1)}{2}|-1_e1_{\rm N}\rangle\langle -1_e1_{\rm N}| + \frac{1-\cos(\theta+\delta\theta_1)}{2}|0_e1_{\rm N}\rangle\langle 0_e1_{\rm N}|\right]\\ +p^s_{0_{\rm N}}&\left[\frac{1+\cos(\theta+\delta\theta_0)}{2}|-1_e0_{\rm N}\rangle\langle -1_e0_{\rm N}| + \frac{1-\cos(\theta+\delta\theta_0)}{2}|0_e0_{\rm N}\rangle\langle 0_e0_{\rm N}|\right]\end{aligned}$$

it can be seen that the electron population is now correlated to the nuclear spin population.

Then the swap gate is applied. This is equal to application of two transition $|0_e1_{\rm N}\rangle \longleftrightarrow |-1_e1_{\rm N}\rangle$, $|0_e1_{\rm N}\rangle \longleftrightarrow |0_e0_{\rm N}\rangle$ and hence the state of the total system becomes

$$\begin{aligned}\rho = p^s_{1_{\rm N}}&\left[\frac{1+\cos(\theta+\delta\theta_1)}{2}|0_e0_{\rm N}\rangle\langle 0_e0_{\rm N}| + \frac{1-\cos(\theta+\delta\theta_1)}{2}|-1_e1_{\rm N}\rangle\langle -1_e1_{\rm N}|\right]\\ +p^s_{0_{\rm N}}&\left[\frac{1+\cos(\theta+\delta\theta_0)}{2}|-1_e0_{\rm N}\rangle\langle -1_e0_{\rm N}| + \frac{1-\cos(\theta+\delta\theta_0)}{2}|0_e1_{\rm N}\rangle\langle 0_e1_{\rm N}|\right]\end{aligned}$$

Finally, the electron is polarized to $|0_e\rangle$, the total state before the first half π pulse becomes

$$\rho = |0_e\rangle\langle 0_e|\otimes\left(\frac{1+p^s_{1_{\rm N}}\cos(\theta+\delta\theta_1)+p^s_{0_{\rm N}}\cos(\theta+\delta\theta_0)}{2}|0_{\rm N}\rangle\langle 0_{\rm N}| + \frac{1-p^s_{1_{\rm N}}\cos(\theta+\delta\theta_1)-p^s_{0_{\rm N}}\cos(\theta+\delta\theta_0)}{2}|1_{\rm N}\rangle\langle 1_{\rm N}|\right)$$

Since $p^s_{1_{\rm N}}$ and $p^s_{0_{\rm N}}$ is steady population, this state should be the same to the state in Eq. (S29). As a result, we obtain a self-consistence equation

$$\begin{aligned}p^s_{1_{\rm N}} &= p^s_{1_{\rm N}}\frac{1-\cos(\theta+\delta\theta_1)}{2} + p^s_{0_{\rm N}}\frac{1-\cos(\theta+\delta\theta_0)}{2}\\ p^s_{0_{\rm N}} &= p^s_{1_{\rm N}}\frac{1+\cos(\theta+\delta\theta_1)}{2} + p^s_{0_{\rm N}}\frac{1+\cos(\theta+\delta\theta_0)}{2}\end{aligned}$$

For ideal case($\delta\theta_m = 0$), the solution is $p^s_{1_N} = (1 - \cos\theta)/2, p^s_{0_N} = (1 + \cos\theta)/2$ and hence the first order signal is the same to the perfect value $p^s_{0_N} - p^s_{1_N} = \cos\theta$ as expected. However, for general case, we find the first order signal to be(using the notation $\delta\theta_0 = 2(\delta_{\text{MW}} + \delta)/\Omega$ and $\delta\theta_1 = 2(\delta_{\text{MW}} - \delta)/\Omega$, δ_{MW} is the detuning of the peaks center to the MW frequency)

$$S_i = p^s_{0_N} - p^s_{1_N} = \frac{\cos(\theta + 2\delta_{\text{MW}}/\Omega)}{1 + p_o \sin(\theta + 2\delta_{\text{MW}}/\Omega)\sin(2\delta/\Omega)}$$

From this formula, the detuning(δ_{MW}) and hyperfine coupling(δ) plays different role. The detuning only introduce a shift to the curve($S_i.vs.\theta$) while the hyperfine coupling(δ) can change the shape of this curve just as observed in the experimental data[see Fig. S14 (a)]. The minimum value arrives at $\theta_{\min} \approx (\pi - 2\delta_{\text{MW}}/\Omega) - 2\delta/\Omega$ and the maximum value arrives at $\theta_{\max} \approx -2\delta_{\text{MW}}/\Omega + 2\delta/\Omega$. As a result, we find

$$\frac{\theta_{\min} - \theta_{\max}}{\pi} = 1 - \frac{4\delta}{\Omega\pi}$$

Hence the left region($[0, \pi)$) would be squeezed or stretched by a ratio $4|\delta|/(\pi\Omega)$ if $\delta > 0$ or $\delta < 0$. For current experimental parameters $\delta = 1.1$MHz(polarized to the subspace $|0_n\rangle, |-1_n\rangle$), $\Omega = 7.29$MHz, we have $(\phi_{\min} - \phi_{max})/\pi = 0.8$, which is also consistent with experimental observation.

Although this theory has capture some mechanism of this abnormal phenomenon, it can't give a comprehensive understanding to all the experimental result. For example, the theory predict the first order signal should be the ideal value when the nitrogen is fully polarized. However, the experimental results show consistence for some MW frequency while inconsistency for other MW frequency[see Fig. S14(b)]. This phenomenon is not well understood and will be investigated in the future. Fortunately, this imperfection can be removed by full polarized the nitrogen and remove the first CNOT in the readout process simultaneously. This is testified for both the nuclear spin signal(Fig. S15) and the RF signal(Fig. S16). As shown in these figures, both the first order signal and second signal show obvious deviation when the nitrogen is not fully polarized[Fig. S15(a) and Fig. S16(a)]. However, as the nitrogen is fully polarized and the first CNOT gate is removed, the signal immediately becomes consistent with the ideal result[Fig. S15(b) and Fig. S16(b)]. In the main text, the amplitude of the second order correlation for ^{13}C signal is measured under such case.

---

[1] Matthias Pfender, Ping Wang, Hitoshi Sumiya, Shinobu Onoda, Wen Yang, Durga Bhaktavatsala Rao Dasari, Philipp Neumann, Xin-Yu Pan, Junichi Isoya, Ren-Bao Liu, and J Wrachtrup. *High-resolution*

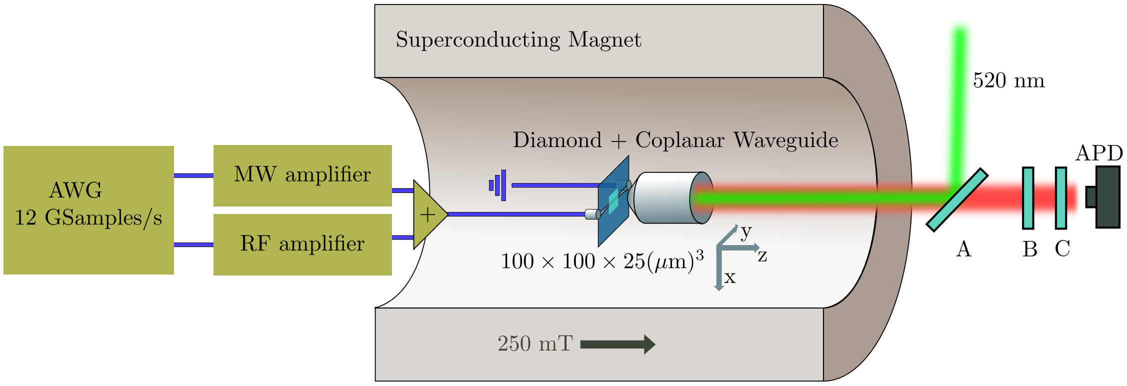

FIG. S1. **Setup**.

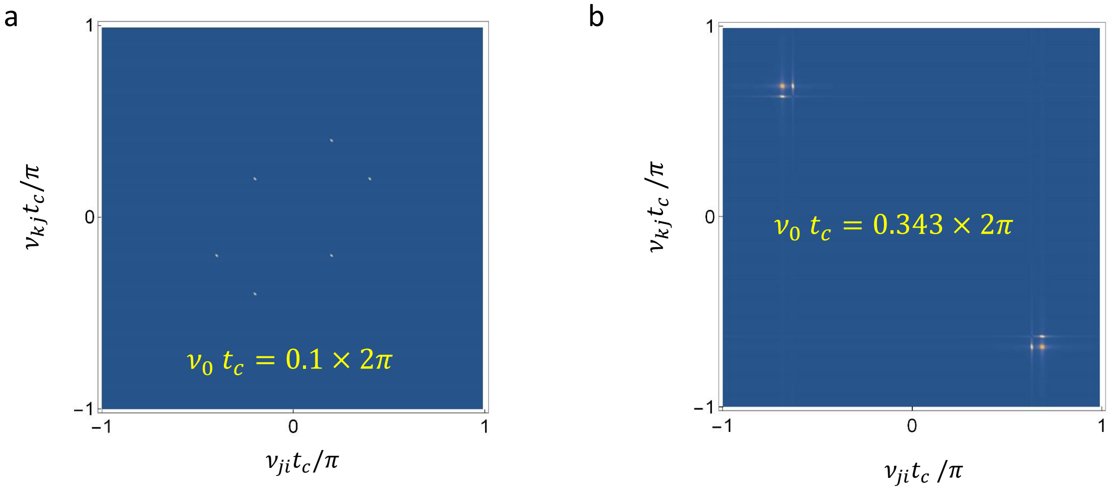

FIG. S2. **Simulation of the Fourier transformation of third order signal for classical AC field. a**. The case of $\nu_0 t_c = 0.1 \times 2\pi$; **b.** The case of experimental parameters $\nu_0 t_c = 0.343 \times 2\pi$.

spectroscopy of single nuclear spins via sequential weak measurements. *Nat. Commun.*, 10(1):594–, 2019.

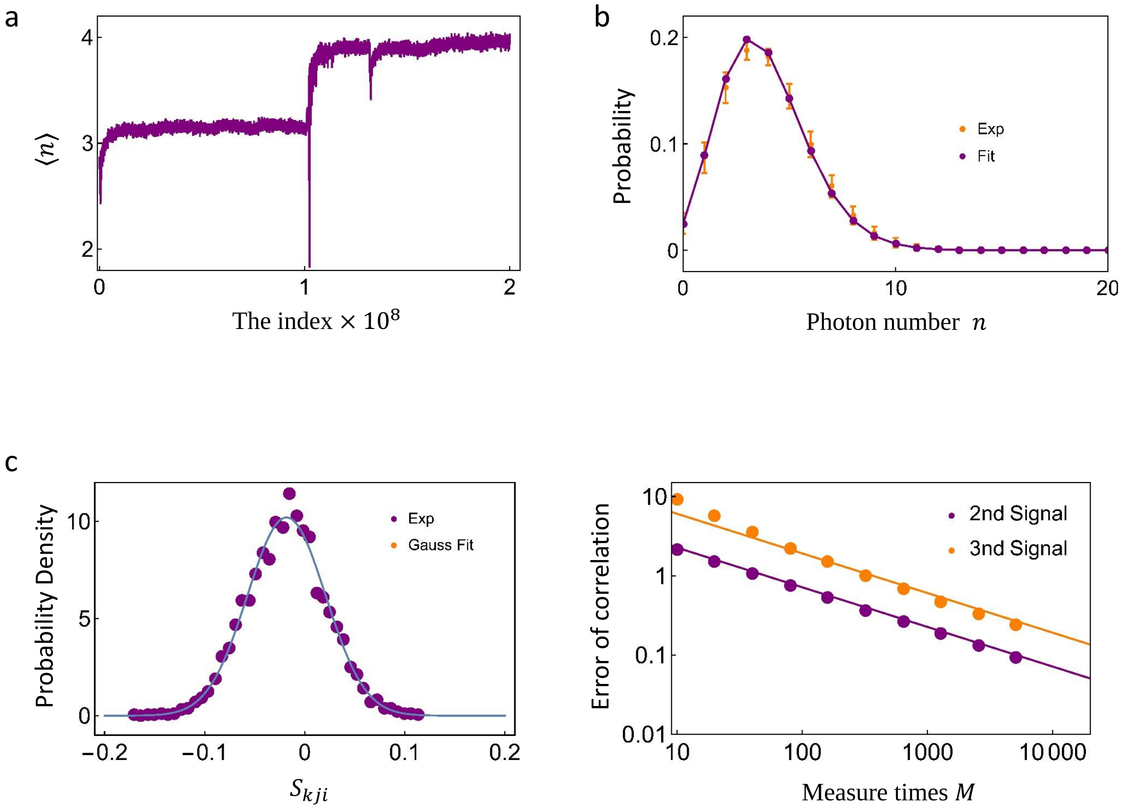

FIG. S3. **Photon counting statistics and Shot noise.** **a**. The trace of the averaged photon number. Each point comes from the averaging of 10^4 times collected photon number; **b.** The distribution of the photon number collected in each measurement cycle; **c.** The distribution of the 3nd signal S_{kji} for fixed time index j, k, i. S_{kji} is calculated from $M = 2\times10^5$ outputs and the probability density function is obtained from 4450 samples. The shot noise of S_{kji} then can be estimated from the width of the probability density function; **d.** The shot noise of 2nd signal S_{ji} and 3nd signal S_{kji} as a function of M(M is the measurement times). The scatters is the experimental result and the line is the theoretical result calculated from Eq.(S28). In the theoretical formula, the averaged photon number $\overline{n}$ is calculated to be about 5.96 from the experimental data. The contrast C is set to be the usual value 0.17(or $n_- = 0.7n_+$).

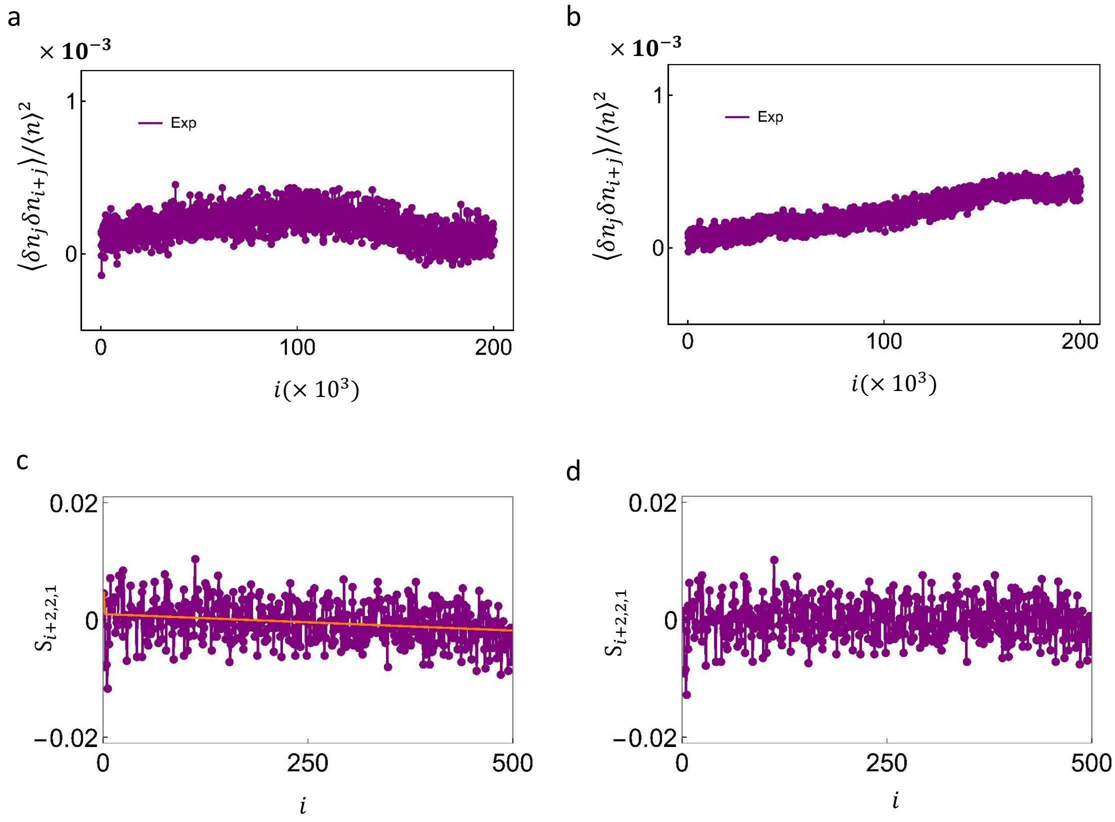

FIG. S4. **The stability of the photon counting statistics:** The normalized cummulant correlation $\langle \delta n_j \delta n_{j+i} \rangle / \langle n \rangle^2$ as a function of interval i for **a**. KDD10 in sample NV1; **b.** KDD5 in sample NV1. **c**. The $S_{i+2,2,1}$ as a function of i; **d**.$S_{i+2,2,1}$vs i where slow linear drift is removed out.

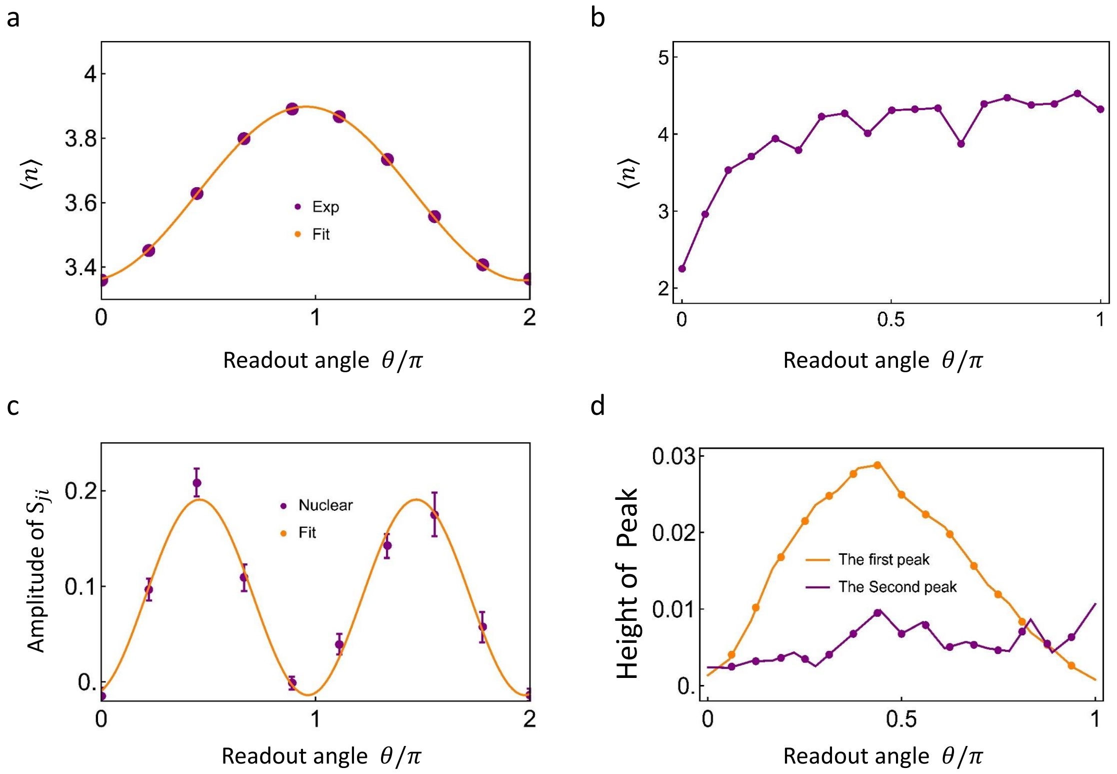

FIG. S5. **The dependence of first and second signal on the readout angle** θ/π**. a**. the first order signal of nuclear spin; **b.** the first order signal of AC magnetic field; **c.** The amplitude of the second order signal of nuclear spin bath; **d.** The peak of the Fourier transform of the second signal for AC magnetic field

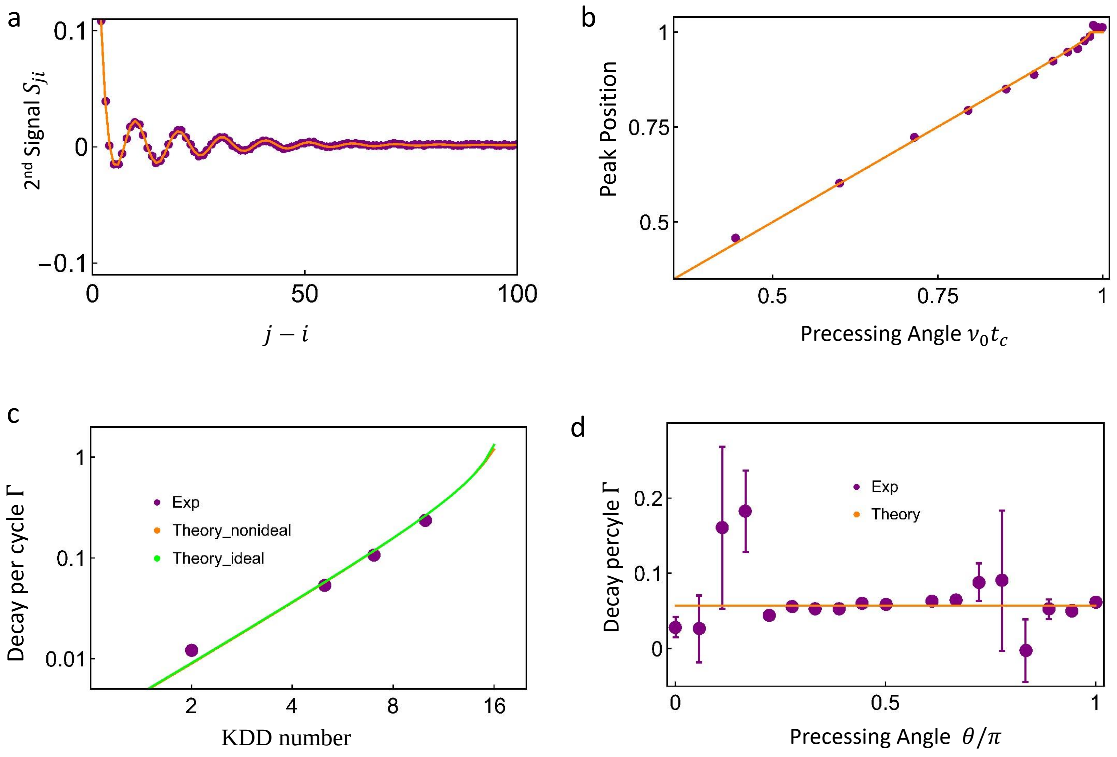

FIG. S6. **The properties of 2nd signal for nuclear spin: a.** The 2nd correlation signal S_{ji} vs the interval $j - i$; **b.** Drag effect: The FFT position as a function of the bare precessing angle $\nu_0 t_c$ in each measurement cycle of the nuclear spin. The red line the theoretical curve for $\alpha = 0.49$; **c**. The total decay per measurement cycle Γ of 2nd signal as a function of the KDD number. The purple scatters is the experimental result and the green line is the simulated result. Experimental data is obtained from Sample II; **d.** Γ as a function of readout angle θ. should

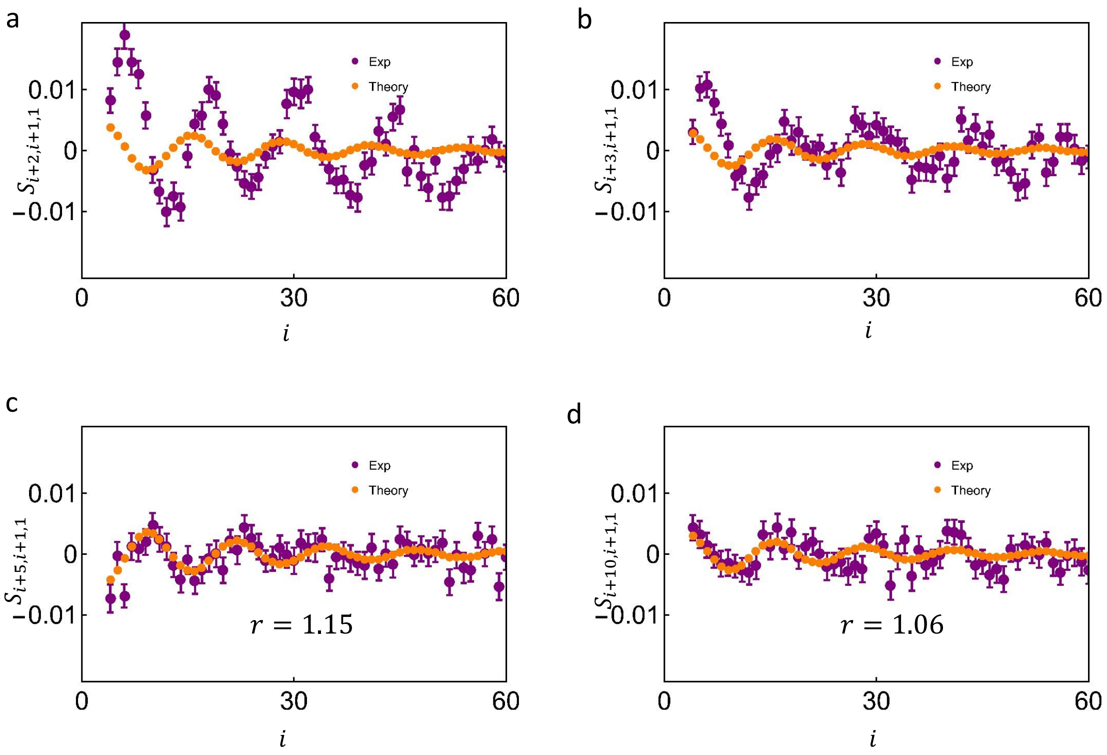

FIG. S7. **The effect of hopping of unknown state on the** S_{kji} **for sample NV2. a** $S_{i+2,i+1,1}$ vs i . Purple scatters is the experimental result of third order signal. Here, the theory result of third order signal(orange scatters) and experimental second order signal(blue scatters) is also shown as a reference; **b**. $S_{i+3,i+1,1}$; **c**. $S_{i+5,i+1,1}$; **d**.$S_{i+10,i+1,1}$. Here $\theta = 53.7$ deg. Here the pulse number is $N_{\mathrm{p}} = 200$. In **c,d**, The amplitude of the theoretical formula is slightly tuned by a factor r to fitting the experimental signal.

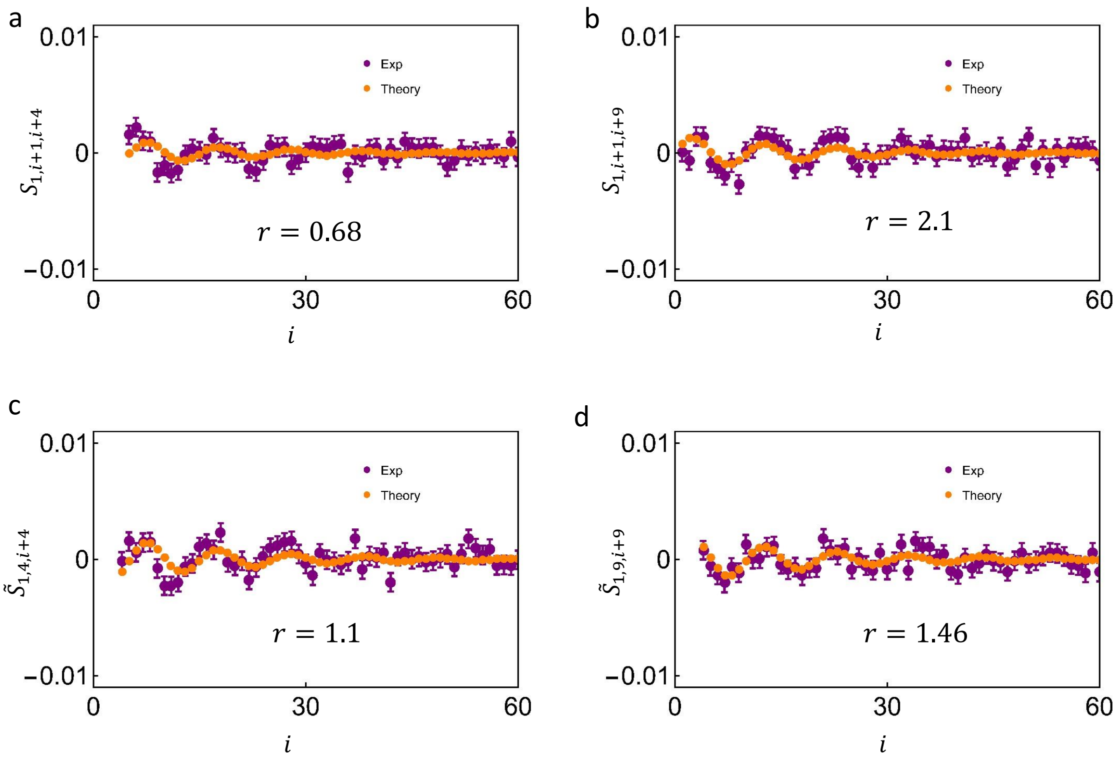

FIG. S8. **The 3nd signal for KDD5 sequence in Sample NV1 .** **a**. The $S_{1,i+1,i+4}$; **b**. The $S_{1,i+1,i+9}(i, 8)$; **c**. The $S_{1,4,i+4}$; **d**. The $S_{1,9,i+9}$ as a function of i. A fast exponential decay and slow photon counting drift is removed out. The amplitude of the theoretical formula is slightly tuned by a factor r to fitting the experimental signal. Here, four data points of the signal in the beginning is removed due to affect of exponential decay signal.

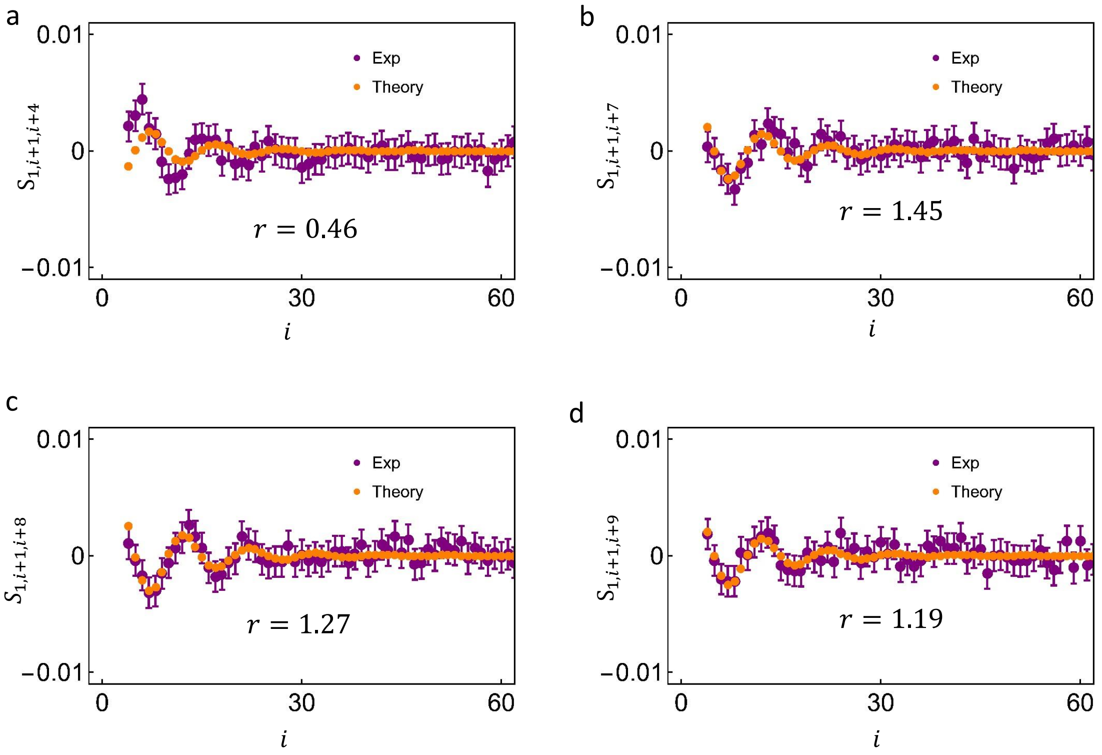

FIG. S9. **The 3nd signal for KDD7 sequence in Sample NV1.** **a**. The $S_{1,i+1,i+4}$; **b**. The $S_{1,i+1,i+7}$; **c**. The $S_{1,i+1,i+8}$; **d**. The $S_{1,i+1,i+9}$ as a function of i. A fast exponential decay and slow photon counting drift is removed out. The amplitude of the theoretical formula is slightly tuned by a factor r to fitting the experimental signal. Here, four data points of the signal in the beginning is removed due to affect of exponential decay signal.

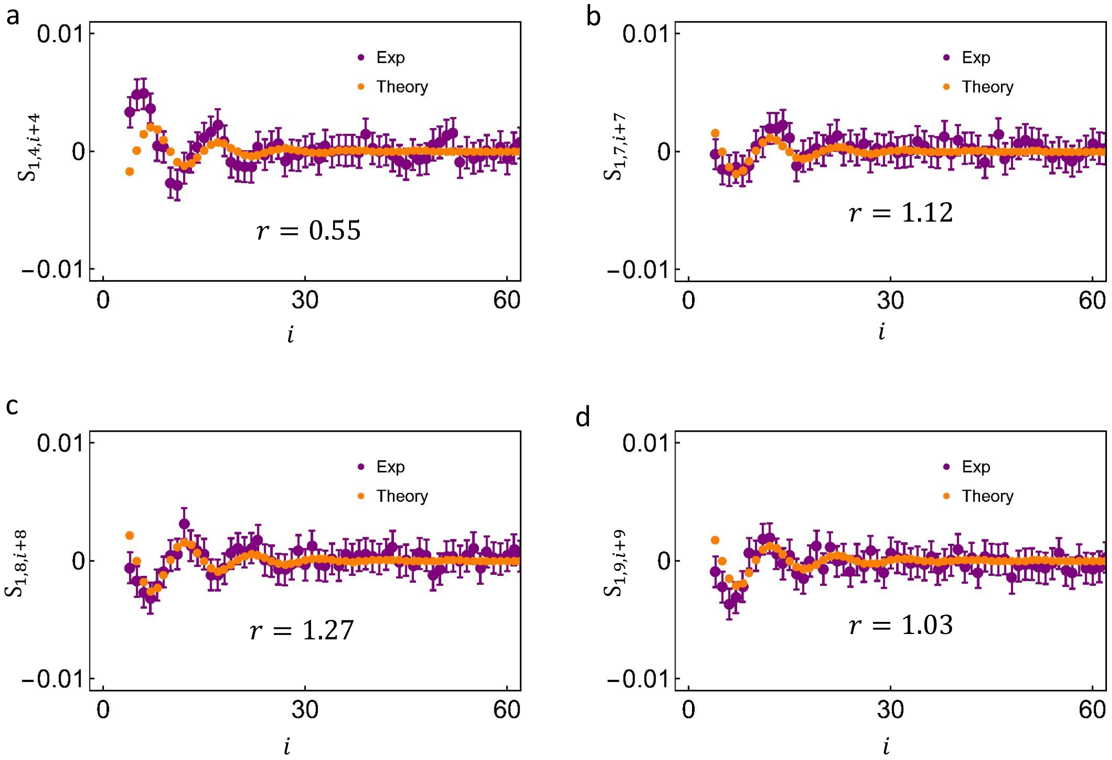

FIG. S10. **The 3nd signal for KDD7 sequence in Sample NV1. a**. The $S_{1,4,i+4}$; **b**. The $S_{1,7,i+7}$; **c**. The $S_{1,8,i+8}$; **d**. The $S_{1,9,i+9}$ as a function of i. A fast exponential decay and slow photon counting drift is removed out. The amplitude of the theoretical formula is slightly tuned by a factor r to fitting the experimental signal. Here, four data points of the signal in the beginning is removed due to affect of exponential decay signal.

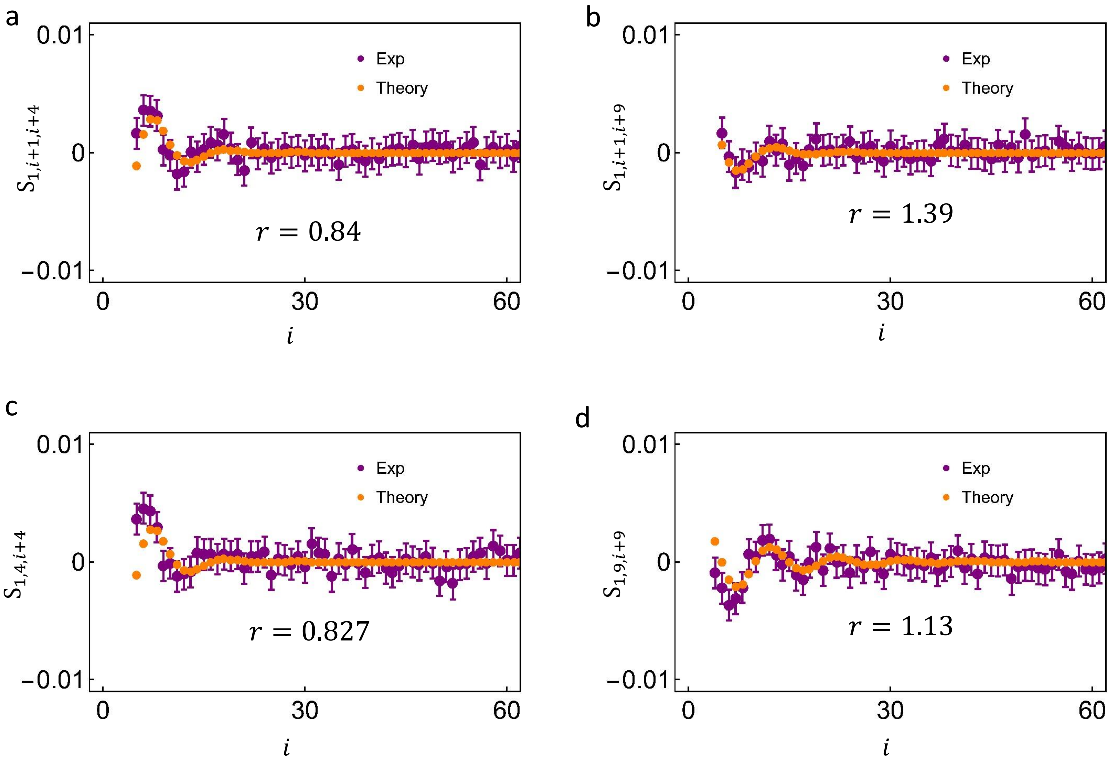

FIG. S11. **The 3nd signal for KDD10 sequence in Sample NV1:** The $S_{1,i+1,i+4}$; **b** The $S_{1,i+1,i+9}$; **c**. The $S_{1,4,i+4}$; **d**. The $S_{1,9,i+9}$ as a function of i. A fast exponential decay and slow photon counting drift is removed out. The amplitude of the theoretical formula is slightly tuned by a factor r to fitting the experimental signal. Here, four data points of the signal in the beginning is removed due to affect of exponential decay signal.

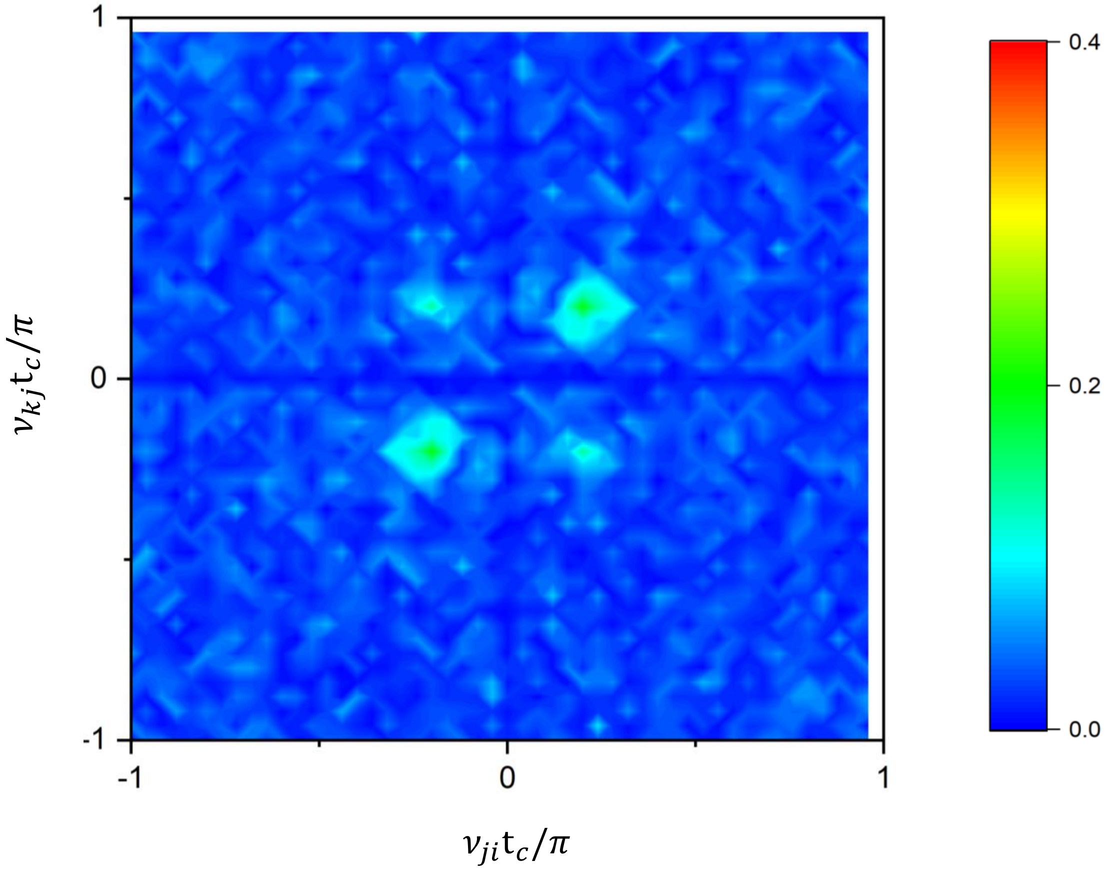

FIG. S12. **The 2D Fourier transform of third order signal for the case of KDD7**.

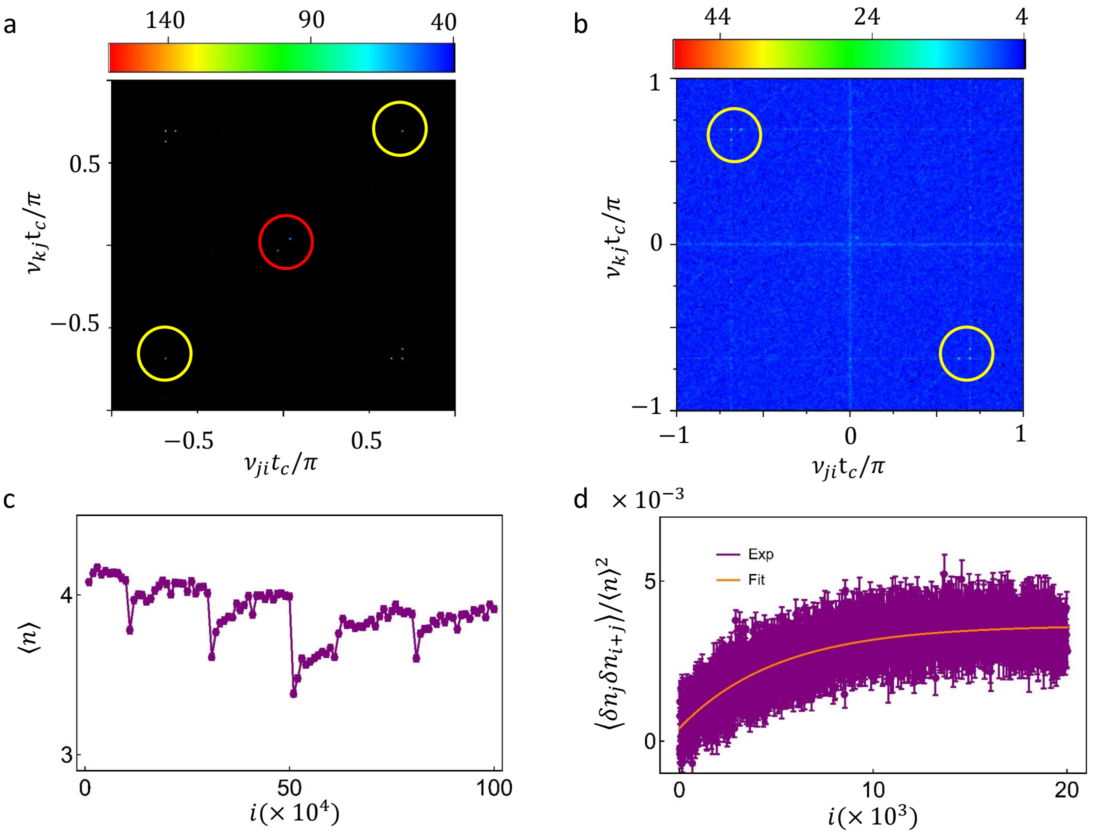

FIG. S13. **The effect of stability of photon counting on non-linear finger-print: a**. FFT of $\tilde{S}_3(i, j)$. $\tilde{S}_3(i, j)$ is calculated from each consecutive 1×10^5 outputs. Then these outputs is averaged for 100 times. **b**. FFT of $\tilde{S}_3(i, j)$. $\tilde{S}_3(i, j)$ is calculated from each consecutive 2.5×10^4 outputs. Then these outputs is averaged for 400 times..

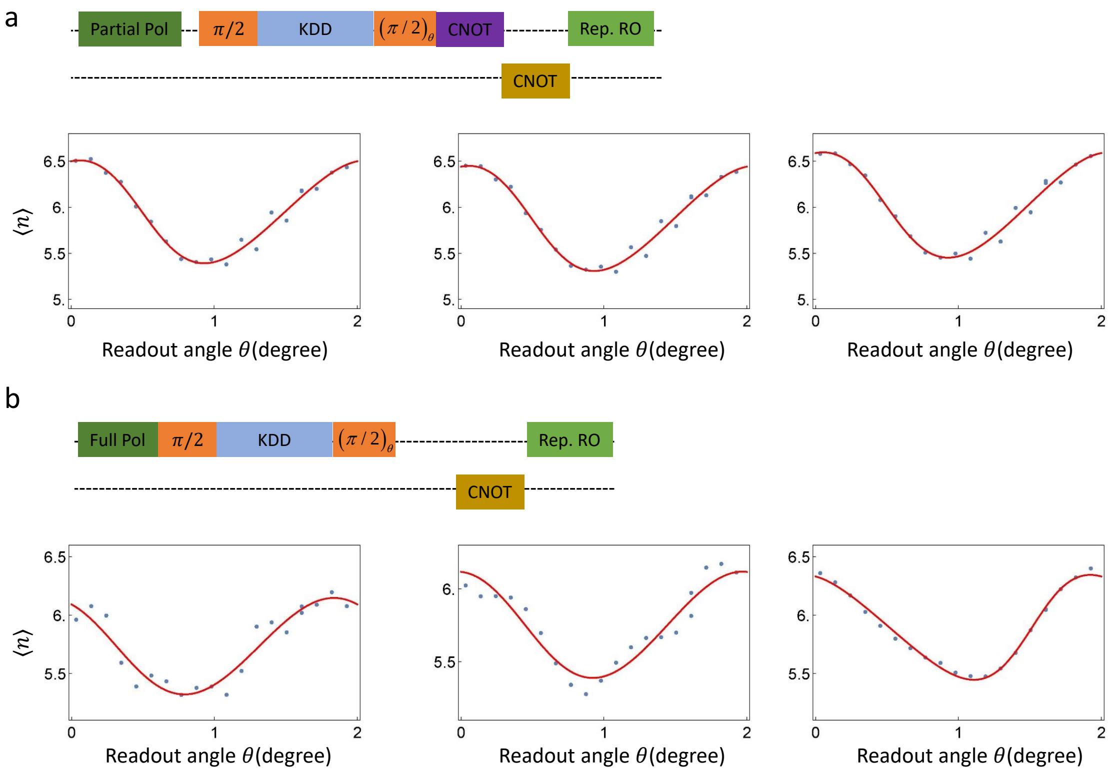

FIG. S14. **The first order signal for partial polarized and full polarized case:** We measure the first order signal as a function readout angle θ under different case: a. The nitrogen is partially polarized to the subspace $|0_\mathrm{N}\rangle, |1_\mathrm{N}\rangle$. From left to right, the MW frequency is chosen to be resonant to $|1_\mathrm{N}\rangle$, the center between $|1_\mathrm{N}\rangle$ and $|0_\mathrm{N}\rangle$, and $|1_\mathrm{N}\rangle$ respectively; b. The nitrogen is fully polarized to level $|0_\mathrm{N}\rangle$. MW is frequency is chosen the same to a.

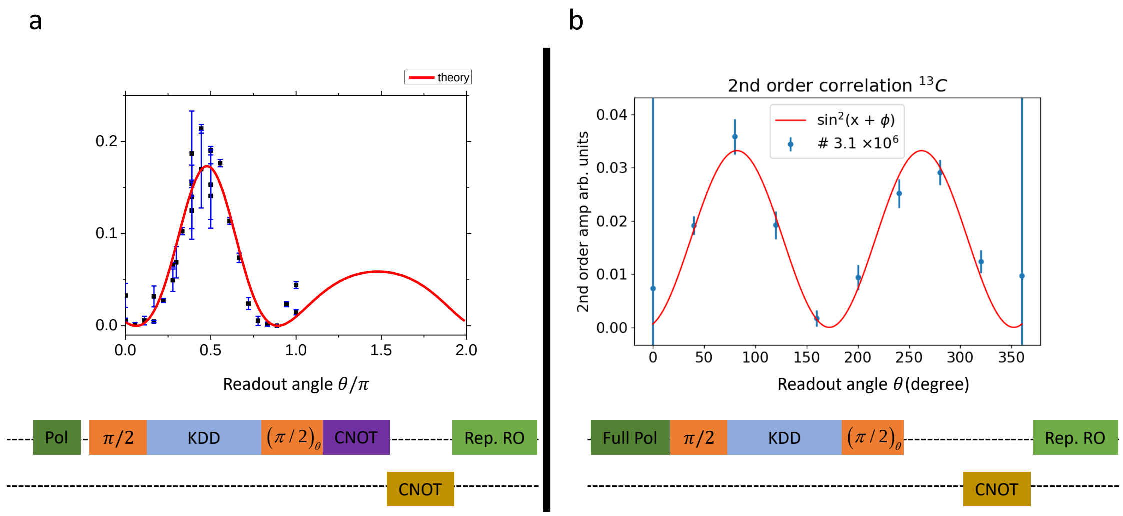

FIG. S15. **The effect of polarization and swap gate to the second order signal of nuclear spin.** The amplitude of second order signal of the ^{13}C vs readout angle θ under two different case: a. The nitrogen is polarized to the subspace of $|0_{\mathrm{N}}\rangle, |1_{\mathrm{N}}\rangle$ and the first CNOT gate in swap process(the purple box) is kept(see the sequence of left graph). The blue scatters is the experimental result while the red curve is the theoretical result when imperfection is considered; b. The nitrogen is fully polarized to the state $|0_{\mathrm{N}}\rangle$ and the first CNOT gate in swap process(the purple box) is removed(see the sequence of right graph).

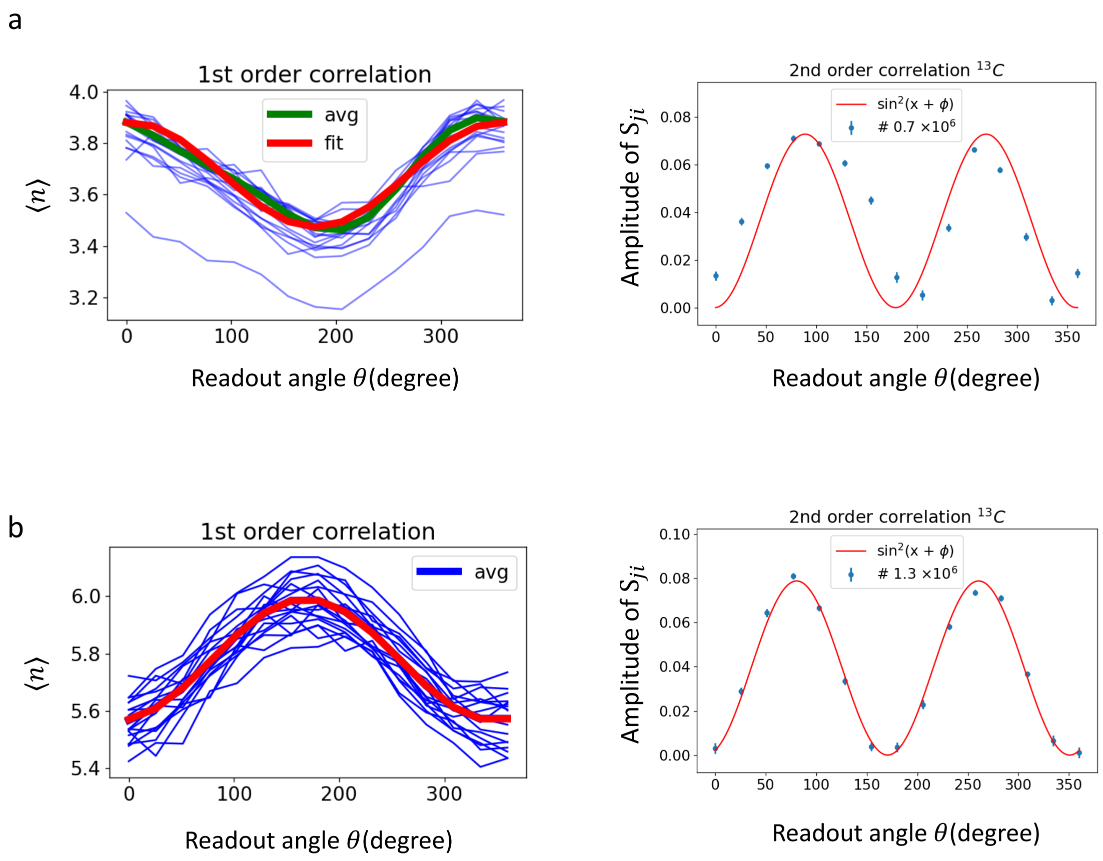

FIG. S16. **The effect of polarization and swap gate to RF signal:** Here we measure the the first order signal(left) and the amplitude of second order signal(right) for classical magnetic field(simulated by RF signal) under different case. a. the first CNOT gate of the swap process in the readout process(see the sequence in the left of Fig. S15) is kept; b. the first CNOT gate is removed(see the sequence in the right of Fig. S15). For both case, the nitrogen is fully polarized to the $|0_N\rangle$.
